# Supplementary material for: State-level prevalence estimates of latent tuberculosis infection in the United States by medical risk factors, demographic characteristics and nativity
Source: PLoS One. 2021 Apr 1;16(4):e0249012. doi: 10.1371/journal.pone.0249012 (PMC8016318; doi:10.1371/journal.pone.0249012)
Supplement: S1 File — (DOCX) [file pone.0249012.s001.docx]

**S1 Appendix. Methods to calculate the** **population-specific annual reactivation rates**

The relative risk of reactivation for different medical risk factors was extracted from an international systematic review and meta-analysis conducted by Yeats (2015). Yeats searched PubMed for papers published in English between 1990 to 2014 with search terms like “TB infection”, “LTBI”, AND “progression” or “reactivation” or “activation.” Citations of the eligible papers were also reviewed for other potential studies. Each population’s relative risk of reactivation (RRR) were extracted. Then, random-effect models were used to estimate the pooled mean and 95% confidence interval limits for the RRR for recent solid organ transplant (vs. others) [Yeats Table C7], HIV infection (vs. HIV negative) [Yeats Table C2], end stage renal disease (ESRD) (vs. no ESRD) [Yeats Table C5], immunosuppressive therapy such as TNF-alpha users (vs. others) [Yeats Table C6], and diabetes mellitus (vs. no diabetes) [Yeats Table C4].

**
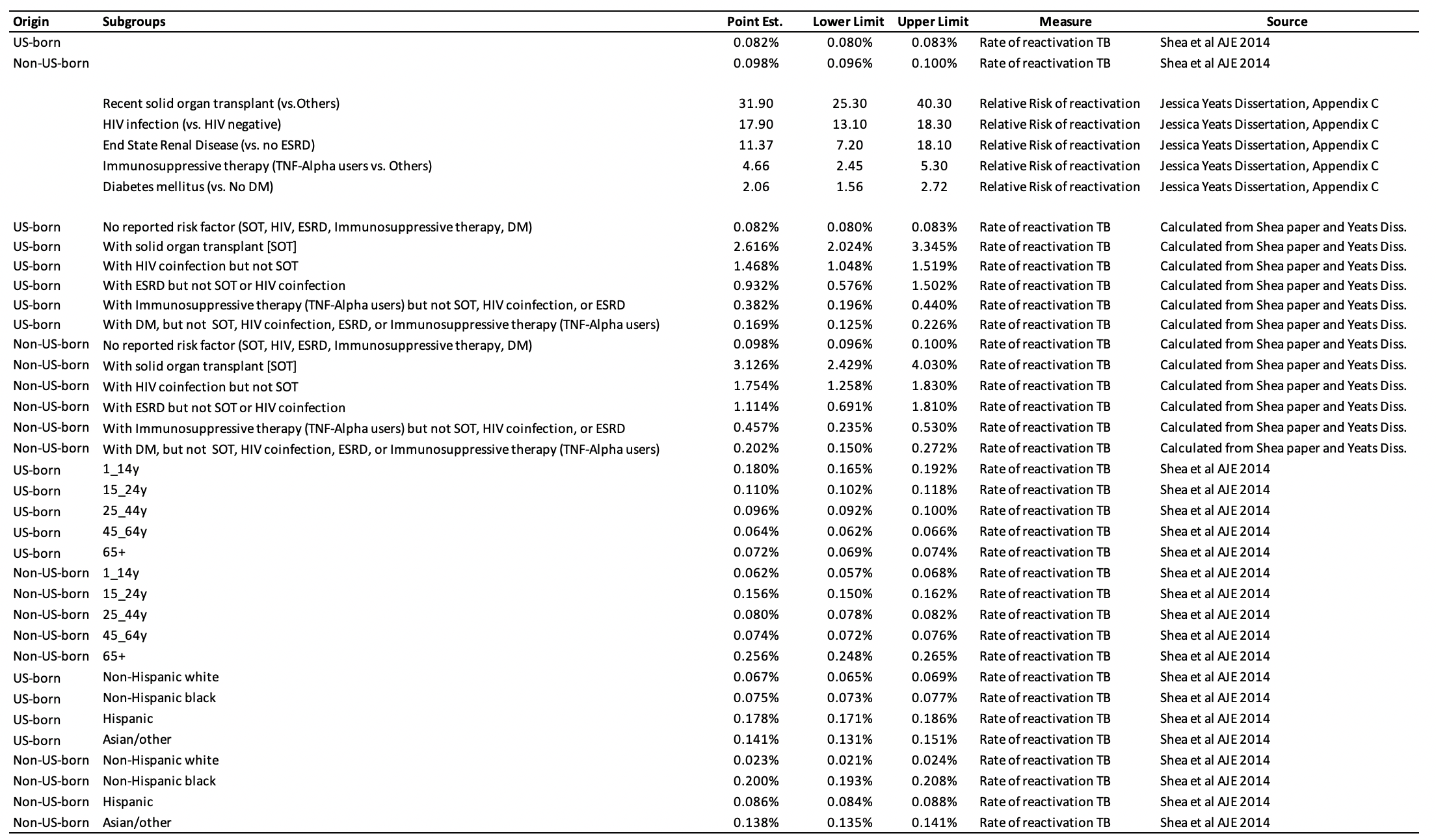
**

We also extracted the reactivation rates calculated by Shea *et al.* (2014) for US-born and non-US–born persons, both in total population and within age group and race/ethnicity groupings. As an example, we calculated the reactivation rate for US-born who are HIV-positive as 0.082% x 17.90 = 1.468% (Lower Limit = 0.080% x 13.10 = 1.048%, Upper Limit = 0.083% x 18.30 = 1.519%).

- Yeats J. Controlling Tuberculosis Among High Risk Populations in Los Angeles. Three Essays (https://www.rand.org/pubs/rgs_dissertations/RGSD356.html) 2015.
- Shea KM, Kammerer JS, Winston CA, Navin TR, Horsburgh CR, Jr. Estimated rate of reactivation of latent tuberculosis infection in the United States, overall and by population subgroup. American Journal of Epidemiology. 2014;179(2):216-25.

**S2 Appendix: the analysis code in R**

# last update 30 Sep 2020 / by Ali

library(readxl)

# change the working directory if needed

setwd("~/Box/CAPE - LTBI/New Model/")

# drop all data

rm(list = ls())

# import reactivation rate

RR <- read_excel("LTBI backcalc new v8 16-July-2019.xlsx", sheet = "RR")

# import TB data

# only import TB data that are not attributed to recent transmission.

# for data with imputed missing, import imputed TB data one at a time and run the rest of the code.

# change the number of imputation in ImputNo, so the results of MI to be saved as different files.

ImputNo <-1

tb <- read.csv("./NEEMA UCSF/tb_dummy.csv")

tb$age<-NA

tb$age[tb$AGE3=="00-04"] <- 0

tb$age[tb$AGE3=="05-14"] <- 0

tb$age[tb$AGE3=="15-24"] <- 1

tb$age[tb$AGE3=="25-44"] <- 2

tb$age[tb$AGE3=="45-64"] <- 3

tb$age[tb$AGE3=="65+"] <- 4

tb$origin[tb$ORIGIN=="USBORN"] <- 0

tb$origin[tb$ORIGIN=="NONUSB"] <- 1

tb$race[tb$RACEHISP=="WHITE"] <- 0

tb$race[tb$RACEHISP=="BLACK"] <- 1

tb$race[tb$RACEHISP=="HISP"] <- 2

tb$race[tb$RACEHISP=="ASIAN"] <- 3

tb$race[tb$RACEHISP=="AMIND"] <- 3

tb$race[tb$RACEHISP=="NAHAW"] <- 3

tb$race[tb$RACEHISP=="MULT"] <- 3

tb$SOT[tb$RISKORGAN=="Y"] <- 1

tb$SOT[tb$RISKORGAN==""] <- 0

tb$HIV<-NA

tb$HIV[tb$HIVSTAT=="NEG"] <- 0

tb$HIV[tb$HIVSTAT=="NOTOFFRD"] <- 0

tb$HIV[tb$HIVSTAT=="IND"] <- 0

tb$HIV[tb$HIVSTAT=="REFUSED"] <- 0

tb$HIV[tb$HIVSTAT=="TDUNK"] <- 0

tb$HIV[tb$HIVSTAT=="UNK"] <- 0

tb$HIV[tb$HIVSTAT=="POS"] <- 1

tb$ESRD[tb$RISKRENAL=="Y"] <- 1

tb$ESRD[tb$RISKRENAL==""] <- 0

tb$IMU[tb$RISKIMMUNO=="Y"] <- 1

tb$IMU[tb$RISKIMMUNO==""] <- 0

tb$DM[tb$RISKDIAB=="Y"] <- 1

tb$DM[tb$RISKDIAB==""] <- 0

tb$rf<-NA

tb$rf[which(tb$SOT==0 & tb$HIV==0 & tb$ESRD==0 & tb$IMU==0 & tb$DM==0)]<-0

tb$rf[which(tb$SOT==1)]<-1

tb$rf[which(tb$SOT==0 & tb$HIV==1)]<-2

tb$rf[which(tb$SOT==0 & tb$HIV==0 & tb$ESRD==1)]<-3

tb$rf[which(tb$SOT==0 & tb$HIV==0 & tb$ESRD==0 & tb$IMU==1 )]<-4

tb$rf[which(tb$SOT==0 & tb$HIV==0 & tb$ESRD==0 & tb$IMU==0 & tb$DM==1)]<-5

ftable(tb$STATE)

ftable(tb$COUNTY)

# Choose your analysis level: State or County

Level<-"State"

if (Level=="State") {

A<-c("US","AK","AL","AR","AZ","CA","CO","CT","DC","DE","FL","GA","HI","IA","ID","IL","IN","KS","KY","LA","MA","MD","ME","MI","MN","MO","MS","MT","NC","ND","NE","NH","NJ","NM","NO","NV","NY","OH","OK","OR","PA","RI","SC","SD","TN","TX","UT","VA","VT","WA","WI","WV","WY")

} else {

A<-c("US","LOS ANGELES","HARRIS","SAN DIEGO","QUEENS","COOK","DALLAS","KINGS","ORANGE","SANTA CLARA","ALAMEDA","MIAMI-DADE","KING","SAN FRANCISCO","MARICOPA","HONOLULU",'HIDALGO',"BRONX","MONTGOMERY","PHILADELPHIA","HENNEPIN")

}

for (S in A) {

#S<-"US"

AreaCode <- S

if (Level=="State") {

if (AreaCode=="US") {

tbArea <- tb

} else {

tbArea<-tb[which(tb$STATE==S),]

}

}

if (Level=="County") {

if (AreaCode=="US") {

tbArea <- tb

} else {

tbArea<-tb[which(tb$COUNTY==S),]

}

}

frqtbRF<-table(tbArea$rf,tbArea$YEAR,tbArea$origin)

frqtbAGE<-table(tbArea$age,tbArea$YEAR,tbArea$origin)

frqtbRACE<-table(tbArea$race,tbArea$YEAR,tbArea$origin)

# add rows with 0 values if there is no TB cases for those cartegories.

frqtbRFDF<-data.frame(unclass(frqtbRF))

RFcat<-c("0","1","2","3","4","5")

RF_TF<-RFcat %in% rownames(frqtbRFDF)

if (RF_TF[1]=="FALSE") {

frqtbRFDF[nrow(frqtbRFDF)+1,] <- 0

rownames(frqtbRFDF)[nrow(frqtbRFDF)]<-"0"

}

if (RF_TF[2]=="FALSE") {

frqtbRFDF[nrow(frqtbRFDF)+1,] <- 0

rownames(frqtbRFDF)[nrow(frqtbRFDF)]<-"1"

}

if (RF_TF[3]=="FALSE") {

frqtbRFDF[nrow(frqtbRFDF)+1,] <- 0

rownames(frqtbRFDF)[nrow(frqtbRFDF)]<-"2"

}

if (RF_TF[4]=="FALSE") {

frqtbRFDF[nrow(frqtbRFDF)+1,] <- 0

rownames(frqtbRFDF)[nrow(frqtbRFDF)]<-"3"

}

if (RF_TF[5]=="FALSE") {

frqtbRFDF[nrow(frqtbRFDF)+1,] <- 0

rownames(frqtbRFDF)[nrow(frqtbRFDF)]<-"4"

}

if (RF_TF[6]=="FALSE") {

frqtbRFDF[nrow(frqtbRFDF)+1,] <- 0

rownames(frqtbRFDF)[nrow(frqtbRFDF)]<-"5"

}

frqtbRFDF<-frqtbRFDF[order(rownames(frqtbRFDF)),]

frqtbAGEDF<-data.frame(unclass(frqtbAGE))

Agecat<-c("0","1","2","3","4")

Age_TF<-Agecat %in% rownames(frqtbAGEDF)

if (Age_TF[1]=="FALSE") {

frqtbAGEDF[nrow(frqtbAGEDF)+1,] <- 0

rownames(frqtbAGEDF)[nrow(frqtbAGEDF)]<-"0"

}

if (Age_TF[2]=="FALSE") {

frqtbAGEDF[nrow(frqtbAGEDF)+1,] <- 0

rownames(frqtbAGEDF)[nrow(frqtbAGEDF)]<-"1"

}

if (Age_TF[3]=="FALSE") {

frqtbAGEDF[nrow(frqtbAGEDF)+1,] <- 0

rownames(frqtbAGEDF)[nrow(frqtbAGEDF)]<-"2"

}

if (Age_TF[4]=="FALSE") {

frqtbAGEDF[nrow(frqtbAGEDF)+1,] <- 0

rownames(frqtbAGEDF)[nrow(frqtbAGEDF)]<-"3"

}

if (Age_TF[5]=="FALSE") {

frqtbAGEDF[nrow(frqtbAGEDF)+1,] <- 0

rownames(frqtbAGEDF)[nrow(frqtbAGEDF)]<-"4"

}

frqtbAGEDF<-frqtbAGEDF[order(rownames(frqtbAGEDF)),]

frqtbRACEDF<-data.frame(unclass(frqtbRACE))

Racecat<-c("0","1","2","3")

Race_TF<-Racecat %in% rownames(frqtbRACEDF)

if (Race_TF[1]=="FALSE") {

frqtbRACEDF[nrow(frqtbRACEDF)+1,] <- 0

rownames(frqtbRACEDF)[nrow(frqtbRACEDF)]<-"0"

}

if (Race_TF[2]=="FALSE") {

frqtbRACEDF[nrow(frqtbRACEDF)+1,] <- 0

rownames(frqtbRACEDF)[nrow(frqtbRACEDF)]<-"1"

}

if (Race_TF[3]=="FALSE") {

frqtbRACEDF[nrow(frqtbRACEDF)+1,] <- 0

rownames(frqtbRACEDF)[nrow(frqtbRACEDF)]<-"2"

}

if (Race_TF[4]=="FALSE") {

frqtbRACEDF[nrow(frqtbRACEDF)+1,] <- 0

rownames(frqtbRACEDF)[nrow(frqtbRACEDF)]<-"3"

}

frqtbRACEDF<-frqtbRACEDF[order(rownames(frqtbRACEDF)),]

D<-rbind(frqtbRFDF,frqtbAGEDF,frqtbRACEDF)

colyears<-c("X2013.0","X2014.0", "X2015.0", "X2016.0", "X2017.0", "X2013.1", "X2014.1", "X2015.1", "X2016.1", "X2017.1")

D_TF<-colyears %in% colnames(D)

if (D_TF[1]=="FALSE") {

D[,ncol(D)+1] <- 0

colnames(D)[ncol(D)]<-"X2013.0"

}

if (D_TF[2]=="FALSE") {

D[,ncol(D)+1] <- 0

colnames(D)[ncol(D)]<-"X2014.0"

}

if (D_TF[3]=="FALSE") {

D[,ncol(D)+1] <- 0

colnames(D)[ncol(D)]<-"X2015.0"

}

if (D_TF[4]=="FALSE") {

D[,ncol(D)+1] <- 0

colnames(D)[ncol(D)]<-"X2016.0"

}

if (D_TF[5]=="FALSE") {

D[,ncol(D)+1] <- 0

colnames(D)[ncol(D)]<-"X2017.0"

}

if (D_TF[6]=="FALSE") {

D[,ncol(D)+1] <- 0

colnames(D)[ncol(D)]<-"X2013.1"

}

if (D_TF[7]=="FALSE") {

D[,ncol(D)+1] <- 0

colnames(D)[ncol(D)]<-"X2014.1"

}

if (D_TF[8]=="FALSE") {

D[,ncol(D)+1] <- 0

colnames(D)[ncol(D)]<-"X2015.1"

}

if (D_TF[9]=="FALSE") {

D[,ncol(D)+1] <- 0

colnames(D)[ncol(D)]<-"X2016.1"

}

if (D_TF[10]=="FALSE") {

D[,ncol(D)+1] <- 0

colnames(D)[ncol(D)]<-"X2017.1"

}

D <- subset(D, select=c("X2013.0","X2014.0", "X2015.0", "X2016.0", "X2017.0", "X2013.1", "X2014.1", "X2015.1", "X2016.1", "X2017.1"))

row.names(D)<-c("No RF",

"With SOT",

"With HIV not SOT",

"With ESRD not SOT or HIV",

"With Immun Tx not SOT, HIV, or ESRD",

"With DM, but not SOT, HIV, ESRD, or Immun Tx",

"1_14y",

"15_24y",

"25_44y",

"45_64y",

"65+",

"Non-Hispanic White",

"Non-Hispanic Black",

"Hispanic",

"Asian/other")

RR1<-cbind(RR[1:6,c("point","ll","ul")],RR[7:12,c("point","ll","ul")])

RR2<-cbind(RR[13:17,c("point","ll","ul")],RR[18:22,c("point","ll","ul")])

RR3<-cbind(RR[23:26,c("point","ll","ul")],RR[27:30,c("point","ll","ul")])

DD<-cbind(D,rbind(RR1,RR2,RR3))

colnames(DD)<-c("X2013.0","X2014.0", "X2015.0", "X2016.0", "X2017.0", "X2013.1", "X2014.1", "X2015.1", "X2016.1", "X2017.1", "point.0", "ll.0", "ul.0", "point.1", "ll.1", "ul.1")

# LTBI est. number for USB from 2013 to 2017

DD$LTBI2013.0<-DD$X2013.0/DD$point.0

DD$LTBI2013ll.0<-DD$X2013.0/DD$ul.0

DD$LTBI2013ul.0<-DD$X2013.0/DD$ll.0

DD$LTBI2014.0<-DD$X2014.0/DD$point.0

DD$LTBI2014ll.0<-DD$X2014.0/DD$ul.0

DD$LTBI2014ul.0<-DD$X2014.0/DD$ll.0

DD$LTBI2015.0<-DD$X2015.0/DD$point.0

DD$LTBI2015ll.0<-DD$X2015.0/DD$ul.0

DD$LTBI2015ul.0<-DD$X2015.0/DD$ll.0

DD$LTBI2016.0<-DD$X2016.0/DD$point.0

DD$LTBI2016ll.0<-DD$X2016.0/DD$ul.0

DD$LTBI2016ul.0<-DD$X2016.0/DD$ll.0

DD$LTBI2017.0<-DD$X2017.0/DD$point.0

DD$LTBI2017ll.0<-DD$X2017.0/DD$ul.0

DD$LTBI2017ul.0<-DD$X2017.0/DD$ll.0

# LTBI est. number for NUSB from 2013 to 2017

DD$LTBI2013.1<-DD$X2013.1/DD$point.1

DD$LTBI2013ll.1<-DD$X2013.1/DD$ul.1

DD$LTBI2013ul.1<-DD$X2013.1/DD$ll.1

DD$LTBI2014.1<-DD$X2014.1/DD$point.1

DD$LTBI2014ll.1<-DD$X2014.1/DD$ul.1

DD$LTBI2014ul.1<-DD$X2014.1/DD$ll.1

DD$LTBI2015.1<-DD$X2015.1/DD$point.1

DD$LTBI2015ll.1<-DD$X2015.1/DD$ul.1

DD$LTBI2015ul.1<-DD$X2015.1/DD$ll.1

DD$LTBI2016.1<-DD$X2016.1/DD$point.1

DD$LTBI2016ll.1<-DD$X2016.1/DD$ul.1

DD$LTBI2016ul.1<-DD$X2016.1/DD$ll.1

DD$LTBI2017.1<-DD$X2017.1/DD$point.1

DD$LTBI2017ll.1<-DD$X2017.1/DD$ul.1

DD$LTBI2017ul.1<-DD$X2017.1/DD$ll.1

# compatibility adj

colSums(DD[c("1_14y","15_24y","25_44y","45_64y","65+"),])

table(DD[c("1_14y","15_24y","25_44y","45_64y","65+"),c("X2013.0")])

DD[16,] = colSums(DD[c("No RF","With SOT","With HIV not SOT","With ESRD not SOT or HIV","With Immun Tx not SOT, HIV, or ESRD","With DM, but not SOT, HIV, ESRD, or Immun Tx"),])

DD[17,] = colSums(DD[c("1_14y","15_24y","25_44y","45_64y","65+"),])

DD[18,] = colSums(DD[c("Non-Hispanic White","Non-Hispanic Black","Hispanic","Asian/other"),])

# RF - replace LL and UL to make the adjustment ratio of point estimates to be used for compatibility correction

DD[c("No RF","With SOT","With HIV not SOT","With ESRD not SOT or HIV","With Immun Tx not SOT, HIV, or ESRD","With DM, but not SOT, HIV, ESRD, or Immun Tx","16"),c("LTBI2013ll.0")] = DD[c("No RF","With SOT","With HIV not SOT","With ESRD not SOT or HIV","With Immun Tx not SOT, HIV, or ESRD","With DM, but not SOT, HIV, ESRD, or Immun Tx","16"),c("LTBI2013.0")]

DD[c("No RF","With SOT","With HIV not SOT","With ESRD not SOT or HIV","With Immun Tx not SOT, HIV, or ESRD","With DM, but not SOT, HIV, ESRD, or Immun Tx","16"),c("LTBI2014ll.0")] = DD[c("No RF","With SOT","With HIV not SOT","With ESRD not SOT or HIV","With Immun Tx not SOT, HIV, or ESRD","With DM, but not SOT, HIV, ESRD, or Immun Tx","16"),c("LTBI2014.0")]

DD[c("No RF","With SOT","With HIV not SOT","With ESRD not SOT or HIV","With Immun Tx not SOT, HIV, or ESRD","With DM, but not SOT, HIV, ESRD, or Immun Tx","16"),c("LTBI2015ll.0")] = DD[c("No RF","With SOT","With HIV not SOT","With ESRD not SOT or HIV","With Immun Tx not SOT, HIV, or ESRD","With DM, but not SOT, HIV, ESRD, or Immun Tx","16"),c("LTBI2015.0")]

DD[c("No RF","With SOT","With HIV not SOT","With ESRD not SOT or HIV","With Immun Tx not SOT, HIV, or ESRD","With DM, but not SOT, HIV, ESRD, or Immun Tx","16"),c("LTBI2016ll.0")] = DD[c("No RF","With SOT","With HIV not SOT","With ESRD not SOT or HIV","With Immun Tx not SOT, HIV, or ESRD","With DM, but not SOT, HIV, ESRD, or Immun Tx","16"),c("LTBI2016.0")]

DD[c("No RF","With SOT","With HIV not SOT","With ESRD not SOT or HIV","With Immun Tx not SOT, HIV, or ESRD","With DM, but not SOT, HIV, ESRD, or Immun Tx","16"),c("LTBI2017ll.0")] = DD[c("No RF","With SOT","With HIV not SOT","With ESRD not SOT or HIV","With Immun Tx not SOT, HIV, or ESRD","With DM, but not SOT, HIV, ESRD, or Immun Tx","16"),c("LTBI2017.0")]

DD[c("No RF","With SOT","With HIV not SOT","With ESRD not SOT or HIV","With Immun Tx not SOT, HIV, or ESRD","With DM, but not SOT, HIV, ESRD, or Immun Tx","16"),c("LTBI2013ll.1")] = DD[c("No RF","With SOT","With HIV not SOT","With ESRD not SOT or HIV","With Immun Tx not SOT, HIV, or ESRD","With DM, but not SOT, HIV, ESRD, or Immun Tx","16"),c("LTBI2013.1")]

DD[c("No RF","With SOT","With HIV not SOT","With ESRD not SOT or HIV","With Immun Tx not SOT, HIV, or ESRD","With DM, but not SOT, HIV, ESRD, or Immun Tx","16"),c("LTBI2014ll.1")] = DD[c("No RF","With SOT","With HIV not SOT","With ESRD not SOT or HIV","With Immun Tx not SOT, HIV, or ESRD","With DM, but not SOT, HIV, ESRD, or Immun Tx","16"),c("LTBI2014.1")]

DD[c("No RF","With SOT","With HIV not SOT","With ESRD not SOT or HIV","With Immun Tx not SOT, HIV, or ESRD","With DM, but not SOT, HIV, ESRD, or Immun Tx","16"),c("LTBI2015ll.1")] = DD[c("No RF","With SOT","With HIV not SOT","With ESRD not SOT or HIV","With Immun Tx not SOT, HIV, or ESRD","With DM, but not SOT, HIV, ESRD, or Immun Tx","16"),c("LTBI2015.1")]

DD[c("No RF","With SOT","With HIV not SOT","With ESRD not SOT or HIV","With Immun Tx not SOT, HIV, or ESRD","With DM, but not SOT, HIV, ESRD, or Immun Tx","16"),c("LTBI2016ll.1")] = DD[c("No RF","With SOT","With HIV not SOT","With ESRD not SOT or HIV","With Immun Tx not SOT, HIV, or ESRD","With DM, but not SOT, HIV, ESRD, or Immun Tx","16"),c("LTBI2016.1")]

DD[c("No RF","With SOT","With HIV not SOT","With ESRD not SOT or HIV","With Immun Tx not SOT, HIV, or ESRD","With DM, but not SOT, HIV, ESRD, or Immun Tx","16"),c("LTBI2017ll.1")] = DD[c("No RF","With SOT","With HIV not SOT","With ESRD not SOT or HIV","With Immun Tx not SOT, HIV, or ESRD","With DM, but not SOT, HIV, ESRD, or Immun Tx","16"),c("LTBI2017.1")]

DD[c("No RF","With SOT","With HIV not SOT","With ESRD not SOT or HIV","With Immun Tx not SOT, HIV, or ESRD","With DM, but not SOT, HIV, ESRD, or Immun Tx","16"),c("LTBI2013ul.0")] = DD[c("No RF","With SOT","With HIV not SOT","With ESRD not SOT or HIV","With Immun Tx not SOT, HIV, or ESRD","With DM, but not SOT, HIV, ESRD, or Immun Tx","16"),c("LTBI2013.0")]

DD[c("No RF","With SOT","With HIV not SOT","With ESRD not SOT or HIV","With Immun Tx not SOT, HIV, or ESRD","With DM, but not SOT, HIV, ESRD, or Immun Tx","16"),c("LTBI2014ul.0")] = DD[c("No RF","With SOT","With HIV not SOT","With ESRD not SOT or HIV","With Immun Tx not SOT, HIV, or ESRD","With DM, but not SOT, HIV, ESRD, or Immun Tx","16"),c("LTBI2014.0")]

DD[c("No RF","With SOT","With HIV not SOT","With ESRD not SOT or HIV","With Immun Tx not SOT, HIV, or ESRD","With DM, but not SOT, HIV, ESRD, or Immun Tx","16"),c("LTBI2015ul.0")] = DD[c("No RF","With SOT","With HIV not SOT","With ESRD not SOT or HIV","With Immun Tx not SOT, HIV, or ESRD","With DM, but not SOT, HIV, ESRD, or Immun Tx","16"),c("LTBI2015.0")]

DD[c("No RF","With SOT","With HIV not SOT","With ESRD not SOT or HIV","With Immun Tx not SOT, HIV, or ESRD","With DM, but not SOT, HIV, ESRD, or Immun Tx","16"),c("LTBI2016ul.0")] = DD[c("No RF","With SOT","With HIV not SOT","With ESRD not SOT or HIV","With Immun Tx not SOT, HIV, or ESRD","With DM, but not SOT, HIV, ESRD, or Immun Tx","16"),c("LTBI2016.0")]

DD[c("No RF","With SOT","With HIV not SOT","With ESRD not SOT or HIV","With Immun Tx not SOT, HIV, or ESRD","With DM, but not SOT, HIV, ESRD, or Immun Tx","16"),c("LTBI2017ul.0")] = DD[c("No RF","With SOT","With HIV not SOT","With ESRD not SOT or HIV","With Immun Tx not SOT, HIV, or ESRD","With DM, but not SOT, HIV, ESRD, or Immun Tx","16"),c("LTBI2017.0")]

DD[c("No RF","With SOT","With HIV not SOT","With ESRD not SOT or HIV","With Immun Tx not SOT, HIV, or ESRD","With DM, but not SOT, HIV, ESRD, or Immun Tx","16"),c("LTBI2013ul.1")] = DD[c("No RF","With SOT","With HIV not SOT","With ESRD not SOT or HIV","With Immun Tx not SOT, HIV, or ESRD","With DM, but not SOT, HIV, ESRD, or Immun Tx","16"),c("LTBI2013.1")]

DD[c("No RF","With SOT","With HIV not SOT","With ESRD not SOT or HIV","With Immun Tx not SOT, HIV, or ESRD","With DM, but not SOT, HIV, ESRD, or Immun Tx","16"),c("LTBI2014ul.1")] = DD[c("No RF","With SOT","With HIV not SOT","With ESRD not SOT or HIV","With Immun Tx not SOT, HIV, or ESRD","With DM, but not SOT, HIV, ESRD, or Immun Tx","16"),c("LTBI2014.1")]

DD[c("No RF","With SOT","With HIV not SOT","With ESRD not SOT or HIV","With Immun Tx not SOT, HIV, or ESRD","With DM, but not SOT, HIV, ESRD, or Immun Tx","16"),c("LTBI2015ul.1")] = DD[c("No RF","With SOT","With HIV not SOT","With ESRD not SOT or HIV","With Immun Tx not SOT, HIV, or ESRD","With DM, but not SOT, HIV, ESRD, or Immun Tx","16"),c("LTBI2015.1")]

DD[c("No RF","With SOT","With HIV not SOT","With ESRD not SOT or HIV","With Immun Tx not SOT, HIV, or ESRD","With DM, but not SOT, HIV, ESRD, or Immun Tx","16"),c("LTBI2016ul.1")] = DD[c("No RF","With SOT","With HIV not SOT","With ESRD not SOT or HIV","With Immun Tx not SOT, HIV, or ESRD","With DM, but not SOT, HIV, ESRD, or Immun Tx","16"),c("LTBI2016.1")]

DD[c("No RF","With SOT","With HIV not SOT","With ESRD not SOT or HIV","With Immun Tx not SOT, HIV, or ESRD","With DM, but not SOT, HIV, ESRD, or Immun Tx","16"),c("LTBI2017ul.1")] = DD[c("No RF","With SOT","With HIV not SOT","With ESRD not SOT or HIV","With Immun Tx not SOT, HIV, or ESRD","With DM, but not SOT, HIV, ESRD, or Immun Tx","16"),c("LTBI2017.1")]

# RACE - replace LL and UL to make the adjustment ratio of point estimates to be used for compatibility correction

DD[c("Non-Hispanic White","Non-Hispanic Black","Hispanic","Asian/other","18"),c("LTBI2013ll.0")] = DD[c("Non-Hispanic White","Non-Hispanic Black","Hispanic","Asian/other","18"),c("LTBI2013.0")]

DD[c("Non-Hispanic White","Non-Hispanic Black","Hispanic","Asian/other","18"),c("LTBI2014ll.0")] = DD[c("Non-Hispanic White","Non-Hispanic Black","Hispanic","Asian/other","18"),c("LTBI2014.0")]

DD[c("Non-Hispanic White","Non-Hispanic Black","Hispanic","Asian/other","18"),c("LTBI2015ll.0")] = DD[c("Non-Hispanic White","Non-Hispanic Black","Hispanic","Asian/other","18"),c("LTBI2015.0")]

DD[c("Non-Hispanic White","Non-Hispanic Black","Hispanic","Asian/other","18"),c("LTBI2016ll.0")] = DD[c("Non-Hispanic White","Non-Hispanic Black","Hispanic","Asian/other","18"),c("LTBI2016.0")]

DD[c("Non-Hispanic White","Non-Hispanic Black","Hispanic","Asian/other","18"),c("LTBI2017ll.0")] = DD[c("Non-Hispanic White","Non-Hispanic Black","Hispanic","Asian/other","18"),c("LTBI2017.0")]

DD[c("Non-Hispanic White","Non-Hispanic Black","Hispanic","Asian/other","18"),c("LTBI2013ll.1")] = DD[c("Non-Hispanic White","Non-Hispanic Black","Hispanic","Asian/other","18"),c("LTBI2013.1")]

DD[c("Non-Hispanic White","Non-Hispanic Black","Hispanic","Asian/other","18"),c("LTBI2014ll.1")] = DD[c("Non-Hispanic White","Non-Hispanic Black","Hispanic","Asian/other","18"),c("LTBI2014.1")]

DD[c("Non-Hispanic White","Non-Hispanic Black","Hispanic","Asian/other","18"),c("LTBI2015ll.1")] = DD[c("Non-Hispanic White","Non-Hispanic Black","Hispanic","Asian/other","18"),c("LTBI2015.1")]

DD[c("Non-Hispanic White","Non-Hispanic Black","Hispanic","Asian/other","18"),c("LTBI2016ll.1")] = DD[c("Non-Hispanic White","Non-Hispanic Black","Hispanic","Asian/other","18"),c("LTBI2016.1")]

DD[c("Non-Hispanic White","Non-Hispanic Black","Hispanic","Asian/other","18"),c("LTBI2017ll.1")] = DD[c("Non-Hispanic White","Non-Hispanic Black","Hispanic","Asian/other","18"),c("LTBI2017.1")]

DD[c("Non-Hispanic White","Non-Hispanic Black","Hispanic","Asian/other","18"),c("LTBI2013ul.0")] = DD[c("Non-Hispanic White","Non-Hispanic Black","Hispanic","Asian/other","18"),c("LTBI2013.0")]

DD[c("Non-Hispanic White","Non-Hispanic Black","Hispanic","Asian/other","18"),c("LTBI2014ul.0")] = DD[c("Non-Hispanic White","Non-Hispanic Black","Hispanic","Asian/other","18"),c("LTBI2014.0")]

DD[c("Non-Hispanic White","Non-Hispanic Black","Hispanic","Asian/other","18"),c("LTBI2015ul.0")] = DD[c("Non-Hispanic White","Non-Hispanic Black","Hispanic","Asian/other","18"),c("LTBI2015.0")]

DD[c("Non-Hispanic White","Non-Hispanic Black","Hispanic","Asian/other","18"),c("LTBI2016ul.0")] = DD[c("Non-Hispanic White","Non-Hispanic Black","Hispanic","Asian/other","18"),c("LTBI2016.0")]

DD[c("Non-Hispanic White","Non-Hispanic Black","Hispanic","Asian/other","18"),c("LTBI2017ul.0")] = DD[c("Non-Hispanic White","Non-Hispanic Black","Hispanic","Asian/other","18"),c("LTBI2017.0")]

DD[c("Non-Hispanic White","Non-Hispanic Black","Hispanic","Asian/other","18"),c("LTBI2013ul.1")] = DD[c("Non-Hispanic White","Non-Hispanic Black","Hispanic","Asian/other","18"),c("LTBI2013.1")]

DD[c("Non-Hispanic White","Non-Hispanic Black","Hispanic","Asian/other","18"),c("LTBI2014ul.1")] = DD[c("Non-Hispanic White","Non-Hispanic Black","Hispanic","Asian/other","18"),c("LTBI2014.1")]

DD[c("Non-Hispanic White","Non-Hispanic Black","Hispanic","Asian/other","18"),c("LTBI2015ul.1")] = DD[c("Non-Hispanic White","Non-Hispanic Black","Hispanic","Asian/other","18"),c("LTBI2015.1")]

DD[c("Non-Hispanic White","Non-Hispanic Black","Hispanic","Asian/other","18"),c("LTBI2016ul.1")] = DD[c("Non-Hispanic White","Non-Hispanic Black","Hispanic","Asian/other","18"),c("LTBI2016.1")]

DD[c("Non-Hispanic White","Non-Hispanic Black","Hispanic","Asian/other","18"),c("LTBI2017ul.1")] = DD[c("Non-Hispanic White","Non-Hispanic Black","Hispanic","Asian/other","18"),c("LTBI2017.1")]

DD[19,] = DD[c("No RF"),]/DD[16,]*DD[17,]

DD[20,] = DD[c("With SOT"),]/DD[16,]*DD[17,]

DD[21,] = DD[c("With HIV not SOT"),]/DD[16,]*DD[17,]

DD[22,] = DD[c("With ESRD not SOT or HIV"),]/DD[16,]*DD[17,]

DD[23,] = DD[c("With Immun Tx not SOT, HIV, or ESRD"),]/DD[16,]*DD[17,]

DD[24,] = DD[c("With DM, but not SOT, HIV, ESRD, or Immun Tx"),]/DD[16,]*DD[17,]

DD[25,] = DD[c("Non-Hispanic White"),]/DD[18,]*DD[17,]

DD[26,] = DD[c("Non-Hispanic Black"),]/DD[18,]*DD[17,]

DD[27,] = DD[c("Hispanic"),]/DD[18,]*DD[17,]

DD[28,] = DD[c("Asian/other"),]/DD[18,]*DD[17,]

row.names(DD)<-c("No RF",

"With SOT",

"With HIV not SOT",

"With ESRD not SOT or HIV",

"With Immun Tx not SOT, HIV, or ESRD",

"With DM, but not SOT, HIV, ESRD, or Immun Tx",

"1_14y",

"15_24y",

"25_44y",

"45_64y",

"65+",

"Non-Hispanic White",

"Non-Hispanic Black",

"Hispanic",

"Asian/other",

"TotalRFs",

"TotalAge",

"TotalRace",

"No RF_C",

"With SOT_C",

"With HIV not SOT_C",

"With ESRD not SOT or HIV_C",

"With Immun Tx not SOT, HIV, or ESRD_C",

"With DM, but not SOT, HIV, ESRD, or Immun Tx_C",

"Non-Hispanic White_C",

"Non-Hispanic Black_C",

"Hispanic_C",

"Asian/other_C")

DD[29,] = colSums(DD[c("No RF_C","With SOT_C","With HIV not SOT_C","With ESRD not SOT or HIV_C","With Immun Tx not SOT, HIV, or ESRD_C","With DM, but not SOT, HIV, ESRD, or Immun Tx_C"),])

DD[30,] = colSums(DD[c("Non-Hispanic White_C","Non-Hispanic Black_C","Hispanic_C","Asian/other_C"),])

rownames(DD)[29]<-"TotalRFs_C"

rownames(DD)[30]<-"TotalRace_C"

DD$LTBI2013<-DD$LTBI2013.0+DD$LTBI2013.1

DD$LTBI2013ll<-DD$LTBI2013ll.0+DD$LTBI2013ll.1

DD$LTBI2013ul<-DD$LTBI2013ul.0+DD$LTBI2013ul.1

DD$LTBI2014<-DD$LTBI2014.0+DD$LTBI2014.1

DD$LTBI2014ll<-DD$LTBI2014ll.0+DD$LTBI2014ll.1

DD$LTBI2014ul<-DD$LTBI2014ul.0+DD$LTBI2014ul.1

DD$LTBI2015<-DD$LTBI2015.0+DD$LTBI2015.1

DD$LTBI2015ll<-DD$LTBI2015ll.0+DD$LTBI2015ll.1

DD$LTBI2015ul<-DD$LTBI2015ul.0+DD$LTBI2015ul.1

DD$LTBI2016<-DD$LTBI2016.0+DD$LTBI2016.1

DD$LTBI2016ll<-DD$LTBI2016ll.0+DD$LTBI2016ll.1

DD$LTBI2016ul<-DD$LTBI2016ul.0+DD$LTBI2016ul.1

DD$LTBI2017<-DD$LTBI2017.0+DD$LTBI2017.1

DD$LTBI2017ll<-DD$LTBI2017ll.0+DD$LTBI2017ll.1

DD$LTBI2017ul<-DD$LTBI2017ul.0+DD$LTBI2017ul.1

DD$Avrg2013to17.0<-rowMeans(DD[,c("LTBI2013.0","LTBI2014.0","LTBI2015.0","LTBI2016.0","LTBI2017.0")])

DD$Avrg2013to17ll.0<-rowMeans(DD[,c("LTBI2013ll.0","LTBI2014ll.0","LTBI2015ll.0","LTBI2016ll.0","LTBI2017ll.0")])

DD$Avrg2013to17ul.0<-rowMeans(DD[,c("LTBI2013ul.0","LTBI2014ul.0","LTBI2015ul.0","LTBI2016ul.0","LTBI2017ul.0")])

DD$Avrg2013to17.1<-rowMeans(DD[,c("LTBI2013.1","LTBI2014.1","LTBI2015.1","LTBI2016.1","LTBI2017.1")])

DD$Avrg2013to17ll.1<-rowMeans(DD[,c("LTBI2013ll.1","LTBI2014ll.1","LTBI2015ll.1","LTBI2016ll.1","LTBI2017ll.1")])

DD$Avrg2013to17ul.1<-rowMeans(DD[,c("LTBI2013ul.1","LTBI2014ul.1","LTBI2015ul.1","LTBI2016ul.1","LTBI2017ul.1")])

DD$Avrg2013to17<-rowMeans(DD[,c("LTBI2013","LTBI2014","LTBI2015","LTBI2016","LTBI2017")])

DD$Avrg2013to17ll<-rowMeans(DD[,c("LTBI2013ll","LTBI2014ll","LTBI2015ll","LTBI2016ll","LTBI2017ll")])

DD$Avrg2013to17ul<-rowMeans(DD[,c("LTBI2013ul","LTBI2014ul","LTBI2015ul","LTBI2016ul","LTBI2017ul")])

DD$Area<-AreaCode

DD$CAT <- row.names(DD)

# save output file as CSV file

#DDshort<-DD[c("No RF_C","With SOT_C","With HIV not SOT_C","With ESRD not SOT or HIV_C","With Immun Tx not SOT, HIV, or ESRD_C","With DM, but not SOT, HIV, ESRD, or Immun Tx_C","TotalRFs_C","1_14y","15_24y","25_44y", "45_64y", "65+","TotalAge","Non-Hispanic White_C","Non-Hispanic Black_C","Hispanic_C","Asian/other_C","TotalRace_C"),c("Area","Avrg2013to17.0","Avrg2013to17ll.0","Avrg2013to17ul.0","Avrg2013to17.1","Avrg2013to17ll.1","Avrg2013to17ul.1","Avrg2013to17","Avrg2013to17ll","Avrg2013to17ul")]

DDshort<-DD[c("No RF_C","With SOT_C","With HIV not SOT_C","With ESRD not SOT or HIV_C","With Immun Tx not SOT, HIV, or ESRD_C","With DM, but not SOT, HIV, ESRD, or Immun Tx_C","TotalRFs_C","No RF","With SOT","With HIV not SOT","With ESRD not SOT or HIV","With Immun Tx not SOT, HIV, or ESRD","With DM, but not SOT, HIV, ESRD, or Immun Tx","TotalRFs","1_14y","15_24y","25_44y", "45_64y", "65+","TotalAge","Non-Hispanic White_C","Non-Hispanic Black_C","Hispanic_C","Asian/other_C","TotalRace_C","Non-Hispanic White","Non-Hispanic Black","Hispanic","Asian/other","TotalRace"),c("CAT","Area","Avrg2013to17.0","Avrg2013to17ll.0","Avrg2013to17ul.0","Avrg2013to17.1","Avrg2013to17ll.1","Avrg2013to17ul.1","Avrg2013to17","Avrg2013to17ll","Avrg2013to17ul")]

DDshort$ErrLL.0<-DDshort$Avrg2013to17.0-DDshort$Avrg2013to17ll.0

DDshort$ErrUL.0<-DDshort$Avrg2013to17ul.0-DDshort$Avrg2013to17.0

DDshort$ErrLL.1<-DDshort$Avrg2013to17.1-DDshort$Avrg2013to17ll.1

DDshort$ErrUL.1<-DDshort$Avrg2013to17ul.1-DDshort$Avrg2013to17.1

DDshort$ErrLL<-DDshort$Avrg2013to17-DDshort$Avrg2013to17ll

DDshort$ErrUL<-DDshort$Avrg2013to17ul-DDshort$Avrg2013to17

write.csv(DDshort,paste0("LTBIest",AreaCode,"_MI",ImputNo,".csv"))

# Store US and States est in one dataframe

if (AreaCode=="US") {

AllDD <- DDshort

} else {

AllDD<-rbind(AllDD,DDshort)

}

}

AllDD[is.na(AllDD)] <- 0

if (Level=="State") {

write.csv(AllDD,paste0("LTBIest_US&States","_MI",ImputNo,".csv"))

}

if (Level=="County") {

write.csv(AllDD,paste0("LTBIest_Counties","_MI",ImputNo,".csv"))

}

# Average the Imputed estimates for US and States

USStates_MI1 <- read.csv("~/Box/NTSS_data_with_RT/October2/LTBIest_US&States_MI1.csv")

USStates_MI2 <- read.csv("~/Box/NTSS_data_with_RT/October2/LTBIest_US&States_MI2.csv")

USStates_MI3 <- read.csv("~/Box/NTSS_data_with_RT/October2/LTBIest_US&States_MI3.csv")

USStates_MI4 <- read.csv("~/Box/NTSS_data_with_RT/October2/LTBIest_US&States_MI4.csv")

USStates_MI5 <- read.csv("~/Box/NTSS_data_with_RT/October2/LTBIest_US&States_MI5.csv")

USStates_MI <- cbind(USStates_MI1,USStates_MI2,USStates_MI3,USStates_MI4,USStates_MI5)

USStates_MI$MI2013to17.0<-rowMeans(USStates_MI[,grepl("Avrg2013to17.0",colnames(USStates_MI))])

USStates_MI$MI2013to17ll.0<-rowMeans(USStates_MI[,grepl("Avrg2013to17ll.0",colnames(USStates_MI))])

USStates_MI$MI2013to17ul.0<-rowMeans(USStates_MI[,grepl("Avrg2013to17ul.0",colnames(USStates_MI))])

USStates_MI$MI2013to17.1<-rowMeans(USStates_MI[,grepl("Avrg2013to17.1",colnames(USStates_MI))])

USStates_MI$MI2013to17ll.1<-rowMeans(USStates_MI[,grepl("Avrg2013to17ll.1",colnames(USStates_MI))])

USStates_MI$MI2013to17ul.1<-rowMeans(USStates_MI[,grepl("Avrg2013to17ul.1",colnames(USStates_MI))])

USStates_MI$MI2013to17<-USStates_MI$MI2013to17.0+USStates_MI$MI2013to17.1

USStates_MI$MI2013to17ll<-USStates_MI$MI2013to17ll.0+USStates_MI$MI2013to17ll.1

USStates_MI$MI2013to17ul<-USStates_MI$MI2013to17ul.0+USStates_MI$MI2013to17ul.1

USStates_MI$ID <- 1:nrow(USStates_MI)

USStates_MI$Area<- toupper(USStates_MI$Area)

USStates_Pop <- read.csv("~/Box/CAPE - LTBI/New Model/PopulationSize.csv")

USStates_Pop$Area<- toupper(USStates_Pop$Area)

USStates_Pop$link<-paste(USStates_Pop$Area,USStates_Pop$CAT,sep="_")

USStates_Pop$link<-gsub(" ", "", USStates_Pop$link, fixed = TRUE)

USStates_MI_Short<-USStates_MI[,c("ID","CAT","Area","MI2013to17.0","MI2013to17ll.0","MI2013to17ul.0","MI2013to17.1","MI2013to17ll.1","MI2013to17ul.1","MI2013to17","MI2013to17ll","MI2013to17ul")]

USStates_MI_Short<-USStates_MI_Short[which(CAT=="TotalAge" | CAT=="With SOT_C" | CAT=="With HIV not SOT_C" | CAT=="With ESRD not SOT or HIV_C" | CAT=="With Immun Tx not SOT, HIV, or ESRD_C" | CAT=="With DM, but not SOT, HIV, ESRD, or Immun Tx_C" | CAT=="No RF_C"| CAT=="1_14y"| CAT=="15_24y"| CAT=="25_44y"| CAT=="45_64y"| CAT=="65+"| CAT=="Non-Hispanic White_C"| CAT=="Non-Hispanic Black_C"| CAT=="Hispanic_C"| CAT=="Asian/other_C"),]

USStates_MI_Short$link<-paste(USStates_MI_Short$Area,USStates_MI_Short$CAT,sep="_")

USStates_MI_Short$link<-gsub(" ", "", USStates_MI_Short$link, fixed = TRUE)

USStates_MI_Short<-merge(USStates_MI_Short,USStates_Pop,by = c("link"), all = TRUE)

# Calculate the PRV

USStates_MI_Short$USBest<-USStates_MI_Short$MI2013to17.0/USStates_MI_Short$USB

USStates_MI_Short$USBestll<-USStates_MI_Short$MI2013to17ll.0/USStates_MI_Short$USB

USStates_MI_Short$USBestul<-USStates_MI_Short$MI2013to17ul.0/USStates_MI_Short$USB

USStates_MI_Short$NUSBest<-USStates_MI_Short$MI2013to17.1/USStates_MI_Short$NUSB

USStates_MI_Short$NUSBestll<-USStates_MI_Short$MI2013to17ll.1/USStates_MI_Short$NUSB

USStates_MI_Short$NUSBestul<-USStates_MI_Short$MI2013to17ul.1/USStates_MI_Short$NUSB

USStates_MI_Short$Totalest<-USStates_MI_Short$MI2013to17/USStates_MI_Short$TOTAL

USStates_MI_Short$Totalestll<-USStates_MI_Short$MI2013to17ll/USStates_MI_Short$TOTAL

USStates_MI_Short$Totalestul<-USStates_MI_Short$MI2013to17ul/USStates_MI_Short$TOTAL

# make columns for Freq. N

USStates_MI_Short$USBltbiN<-paste(format(round(USStates_MI_Short$MI2013to17.0,digits=0),big.mark=",",scientific=FALSE)," (",format(round(USStates_MI_Short$MI2013to17ll.0,digits=0),big.mark=",",scientific=FALSE)," to ",format(round(USStates_MI_Short$MI2013to17ul.0,digits=0),big.mark=",",scientific=FALSE),")")

USStates_MI_Short$USBltbiN<-gsub(" ", "", USStates_MI_Short$USBltbiN, fixed = TRUE)

USStates_MI_Short$USBltbiN<-gsub("to", " to ", USStates_MI_Short$USBltbiN, fixed = TRUE)

USStates_MI_Short$USBltbiN<-gsub("(", " (", USStates_MI_Short$USBltbiN, fixed = TRUE)

USStates_MI_Short$NUSBltbiN<-paste(format(round(USStates_MI_Short$MI2013to17.1,digits=0),big.mark=",",scientific=FALSE)," (",format(round(USStates_MI_Short$MI2013to17ll.1,digits=0),big.mark=",",scientific=FALSE)," to ",format(round(USStates_MI_Short$MI2013to17ul.1,digits=0),big.mark=",",scientific=FALSE),")")

USStates_MI_Short$NUSBltbiN<-gsub(" ", "", USStates_MI_Short$NUSBltbiN, fixed = TRUE)

USStates_MI_Short$NUSBltbiN<-gsub("to", " to ", USStates_MI_Short$NUSBltbiN, fixed = TRUE)

USStates_MI_Short$NUSBltbiN<-gsub("(", " (", USStates_MI_Short$NUSBltbiN, fixed = TRUE)

USStates_MI_Short$TotltbiN<-paste(format(round(USStates_MI_Short$MI2013to17,digits=0),big.mark=",",scientific=FALSE)," (",format(round(USStates_MI_Short$MI2013to17ll,digits=0),big.mark=",",scientific=FALSE)," to ",format(round(USStates_MI_Short$MI2013to17ul,digits=0),big.mark=",",scientific=FALSE),")")

USStates_MI_Short$TotltbiN<-gsub(" ", "", USStates_MI_Short$TotltbiN, fixed = TRUE)

USStates_MI_Short$TotltbiN<-gsub("to", " to ", USStates_MI_Short$TotltbiN, fixed = TRUE)

USStates_MI_Short$TotltbiN<-gsub("(", " (", USStates_MI_Short$TotltbiN, fixed = TRUE)

# make columns for % pervalence

USStates_MI_Short$USBltbiP<-paste(format(round(USStates_MI_Short$USBest*100,digits=1),scientific=FALSE)," (",format(round(USStates_MI_Short$USBestll*100,digits=1),scientific=FALSE)," to ",format(round(USStates_MI_Short$USBestul*100,digits=1),scientific=FALSE),")")

USStates_MI_Short$USBltbiP<-gsub(" ", "", USStates_MI_Short$USBltbiP, fixed = TRUE)

USStates_MI_Short$USBltbiP<-gsub("to", " to ", USStates_MI_Short$USBltbiP, fixed = TRUE)

USStates_MI_Short$USBltbiP<-gsub("(", " (", USStates_MI_Short$USBltbiP, fixed = TRUE)

USStates_MI_Short$NUSBltbiP<-paste(format(round(USStates_MI_Short$NUSBest*100,digits=1),scientific=FALSE)," (",format(round(USStates_MI_Short$NUSBestll*100,digits=1),scientific=FALSE)," to ",format(round(USStates_MI_Short$NUSBestul*100,digits=1),scientific=FALSE),")")

USStates_MI_Short$NUSBltbiP<-gsub(" ", "", USStates_MI_Short$NUSBltbiP, fixed = TRUE)

USStates_MI_Short$NUSBltbiP<-gsub("to", " to ", USStates_MI_Short$NUSBltbiP, fixed = TRUE)

USStates_MI_Short$NUSBltbiP<-gsub("(", " (", USStates_MI_Short$NUSBltbiP, fixed = TRUE)

USStates_MI_Short$TotltbiP<-paste(format(round(USStates_MI_Short$Totalest*100,digits=1),scientific=FALSE)," (",format(round(USStates_MI_Short$Totalestll*100,digits=1),scientific=FALSE)," to ",format(round(USStates_MI_Short$Totalestul*100,digits=1),scientific=FALSE),")")

USStates_MI_Short$TotltbiP<-gsub(" ", "", USStates_MI_Short$TotltbiP, fixed = TRUE)

USStates_MI_Short$TotltbiP<-gsub("to", " to ", USStates_MI_Short$TotltbiP, fixed = TRUE)

USStates_MI_Short$TotltbiP<-gsub("(", " (", USStates_MI_Short$TotltbiP, fixed = TRUE)

write.csv(USStates_MI_Short,paste0("USStates_MI_","FINAL",".csv"))

# make maps

##################

library(usmap)

library(ggplot2)

library(scales)

# read your LTBI FINAL data

myData <- read.csv("~/Box/CAPE - LTBI/New Model/USStates_MI_FINAL.csv")

StateNames<-read.csv("~/Box/CAPE - LTBI/Maps/StateNames.csv")

myData <- merge(myData, StateNames, by.x="Area.x", by.y="Code", duplicateGeoms = TRUE )

names(myData)[names(myData) == "State"] <- "state"

#states_map$states<-states_map$full

#myData <- left_join(states_map, D, by = "states")

myData<-within(myData,{

Gr_U <- NA

Gr_U[USBest < 0.005] <- 1

Gr_U[USBest >= 0.005 & USBest < 0.01] <- 2

Gr_U[USBest >= 0.01 & USBest < 0.02] <- 3

Gr_U[USBest >= 0.02] <- 4 })

myData<-within(myData,{

Gr_N <- NA

Gr_N[NUSBest < 0.05] <- 1

Gr_N[NUSBest >= 0.05 & NUSBest < 0.10] <- 2

Gr_N[NUSBest >= 0.10 & NUSBest < 0.15] <- 3

Gr_N[NUSBest >= 0.15] <- 4 })

myData<-within(myData,{

Gr_T <- NA

Gr_T[Totalest < 0.01] <- 1

Gr_T[Totalest >= 0.01 & Totalest < 0.02] <- 2

Gr_T[Totalest >= 0.02 & Totalest < 0.03] <- 3

Gr_T[Totalest >= 0.03] <- 4 })

myData<-within(myData,{

CAT <- NA

CAT[CAT.x == "TotalAge"] <- "People of all ages"

CAT[CAT.x == "1_14y"] <- "1 to 14 years"

CAT[CAT.x == "15_24y"] <- "15 to 24 years"

CAT[CAT.x == "25_44y"] <- "25 to 44 years"

CAT[CAT.x == "45_64y"] <- "45 to 64 years"

CAT[CAT.x == "65+"] <- "65+ years"

CAT[CAT.x == "Non-Hispanic White_C"] <- "Non-Hispanic White"

CAT[CAT.x == "Non-Hispanic Black_C"] <- "Non-Hispanic Black"

CAT[CAT.x == "Hispanic_C"] <- "Hispanic"

CAT[CAT.x == "Asian/other_C"] <- "Asian/other"

})

head(myData)

subg<-c("People of all ages","1 to 14 years","15 to 24 years","25 to 44 years","45 to 64 years","65+ years","Non-Hispanic White",

"Non-Hispanic Black","Hispanic","Asian/other")

for (S in subg) {

#S<-"TotalAge"

myData2<-myData[which(myData$CAT== S),]

myData2$USBest[which(myData2$USBest>1)] <-1

myData2$NUSBest[which(myData2$NUSBest>1)] <-1

myData2$Totalest[which(myData2$Totalest>1)] <-1

# Freq tables

print(S)

print("USB States Frq by cutoff 0% to <0.5% / 0.5% to <1% / 1% to <2% / 2%+")

print(ftable(myData2$Gr_U))

print("NUSB States Frq by cutoff [ 0% to <5% / 5% to <10% / 10% to <15% / 15% +")

print(ftable(myData2$Gr_N))

print("Total States Frq by cutoff [ 0% to <1% / 1% to <2% / 2% to <3% / 3% +")

print(ftable(myData2$Gr_T))

# US-born

Map_USB <- plot_usmap(data = myData2, values = "USBest", color = "grey23") +

scale_fill_continuous(low = "White", high = "dark blue", breaks = quantile(myData2$USBest, probs = c(0, 0.25,0.5,0.75,1), na.rm = TRUE) , labels = label_percent()) +

theme(legend.title = element_blank(),legend.position="right", plot.title = element_text(hjust=0.5,size=12, face='bold')) +

ggtitle(paste("US-born","(",S,")"))

Map_USB

pdf(paste("Map_USB",gsub("/", "&", S, fixed = TRUE),".pdf"))

print(Map_USB)

dev.off()

# non-US-born

Map_NUSB <- plot_usmap(data = myData2, values = "NUSBest", color = "grey23") +

scale_fill_continuous(low = "White", high = "dark green", breaks = quantile(myData2$NUSBest, probs = c(0, 0.25,0.5,0.75,1), na.rm = TRUE) , labels = label_percent()) +

theme(legend.title = element_blank(),legend.position="right", plot.title = element_text(hjust=0.5,size=12, face='bold')) +

ggtitle(paste("non-US-born","(",S,")"))

Map_NUSB

pdf(paste("Map_NUSB",gsub("/", "&", S, fixed = TRUE),".pdf"))

print(Map_NUSB)

dev.off()

# Total Pop

Map_Tot <- plot_usmap(data = myData2, values = "Totalest", color = "grey23") +

scale_fill_continuous(low = "White", high = "red", breaks = quantile(myData2$Totalest, probs = c(0, 0.25,0.5,0.75,1), na.rm = TRUE) , labels = label_percent()) +

theme(legend.title = element_blank(),legend.position="right", plot.title = element_text(hjust=0.5,size=12, face='bold')) +

ggtitle(paste("Total","(",S,")"))

Map_Tot

pdf(paste("Map_Total",gsub("/", "&", S, fixed = TRUE),".pdf"))

print(Map_Tot)

dev.off()

}

**S3 Appendix: the structure of “LTBI backcalc RR data.xlsx”**

**
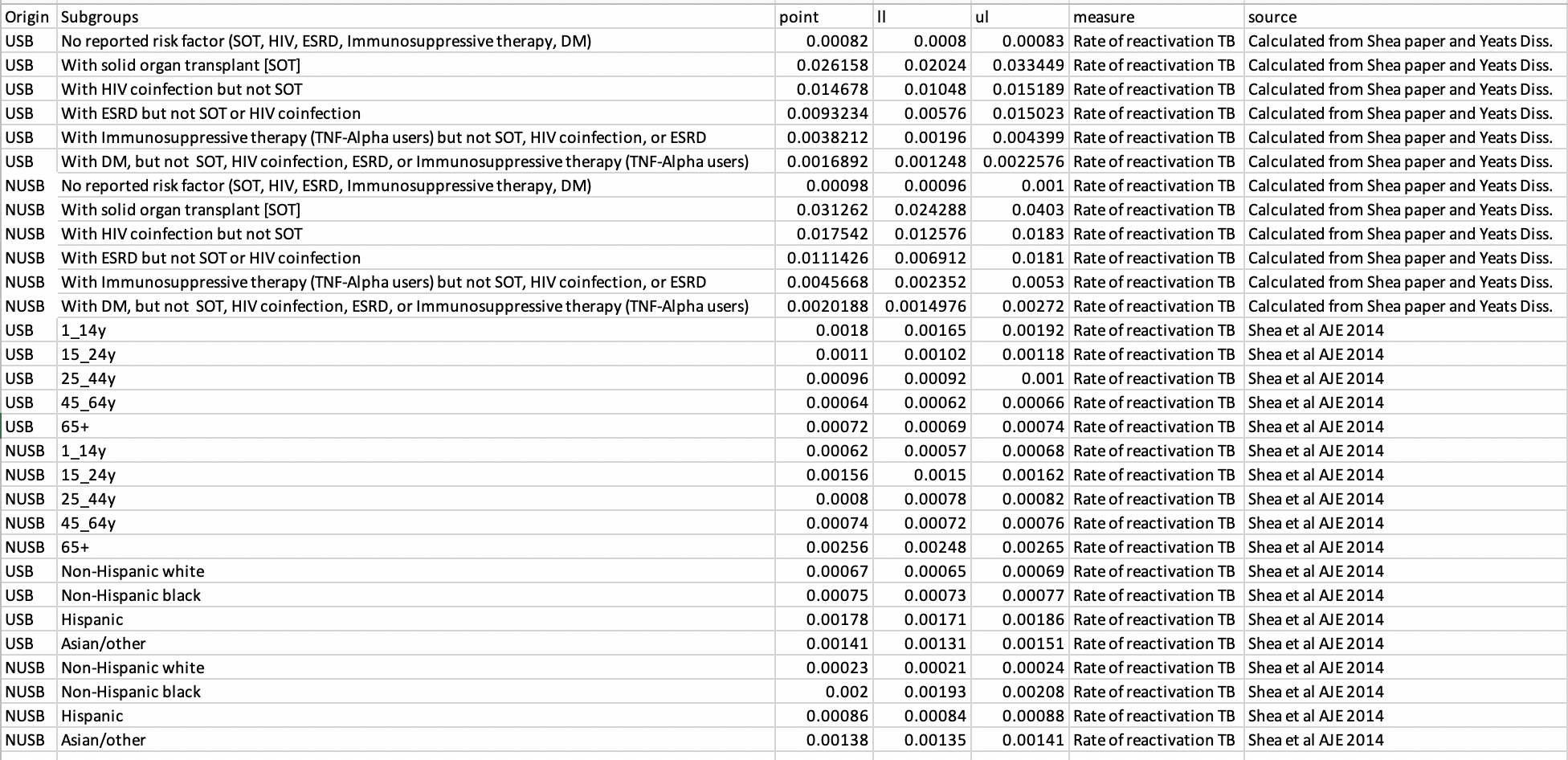
**

**S4 Appendix: The structure of tb_dummyRT.csv**

**
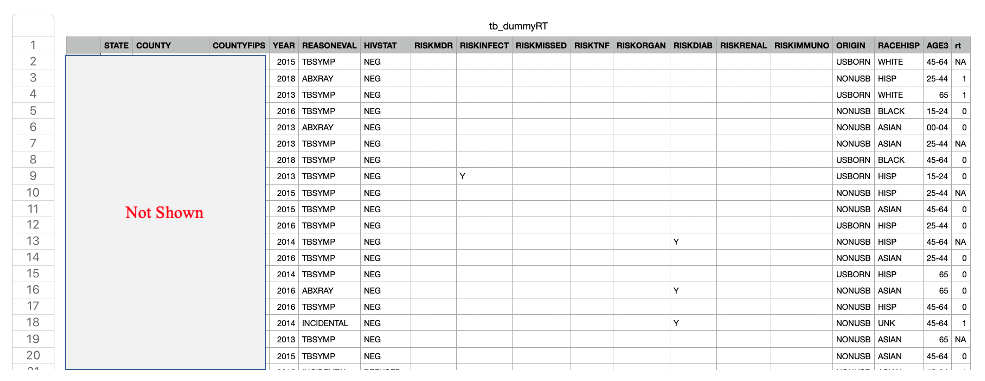
**

**S1 Table. Estimated prevalence and number of persons living with latent tuberculosis infection by medical risk factor, age group, and race/ethnicity groupings, stratified by nativity and in total population, in 50 US states and District of Columbia, 2015**

*** Table is in the Excel file attached to the manuscript “sheet: S1 Table ***

**S1 Fig. Estimated prevalence of latent tuberculosis infection by state and age, United States, 2015. (**The numbers in the legend are minimum, 25%, 50%, 75% quintiles, and maximum values.)*.* Software and source: open-source R and “usmap” package were used to create the maps. Both R software and the “usmap” package are license under GPL-3 | file LICENSE and free to use. [<https://www.r-project.org/Licenses/>; <https://cran.r-project.org/web/packages/usmap/usmap.pdf>]

| 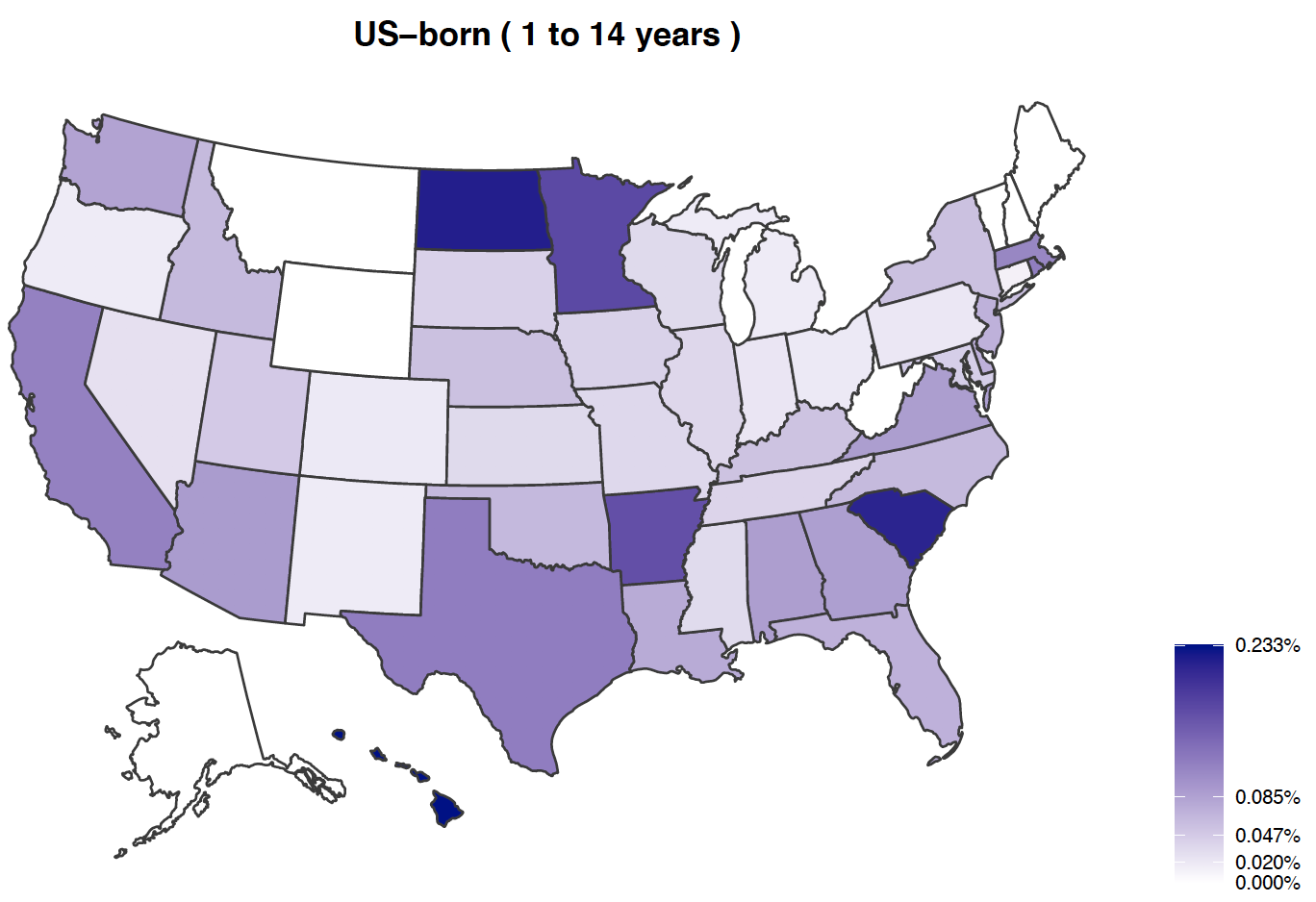 | 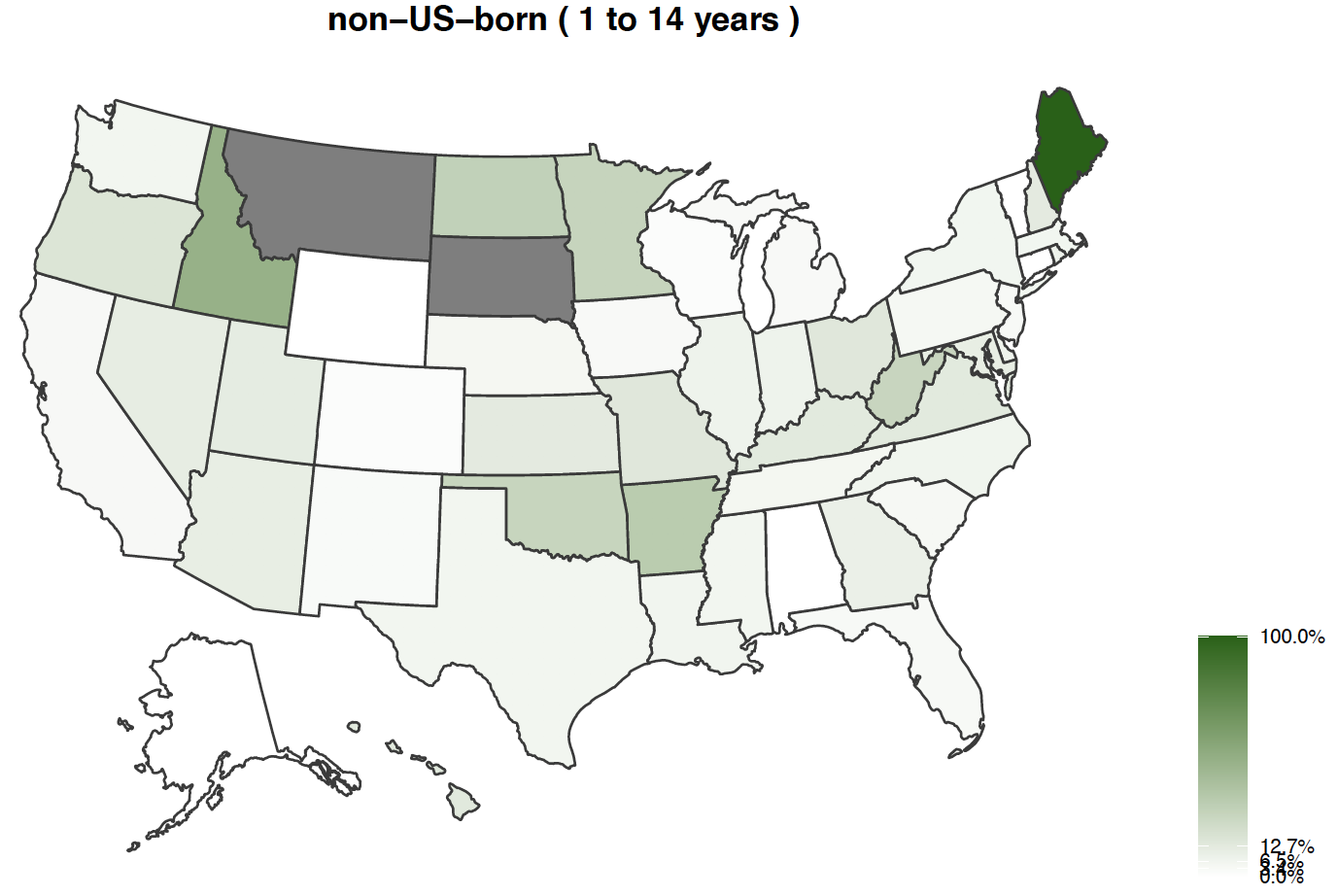 |
| --- | --- |
| **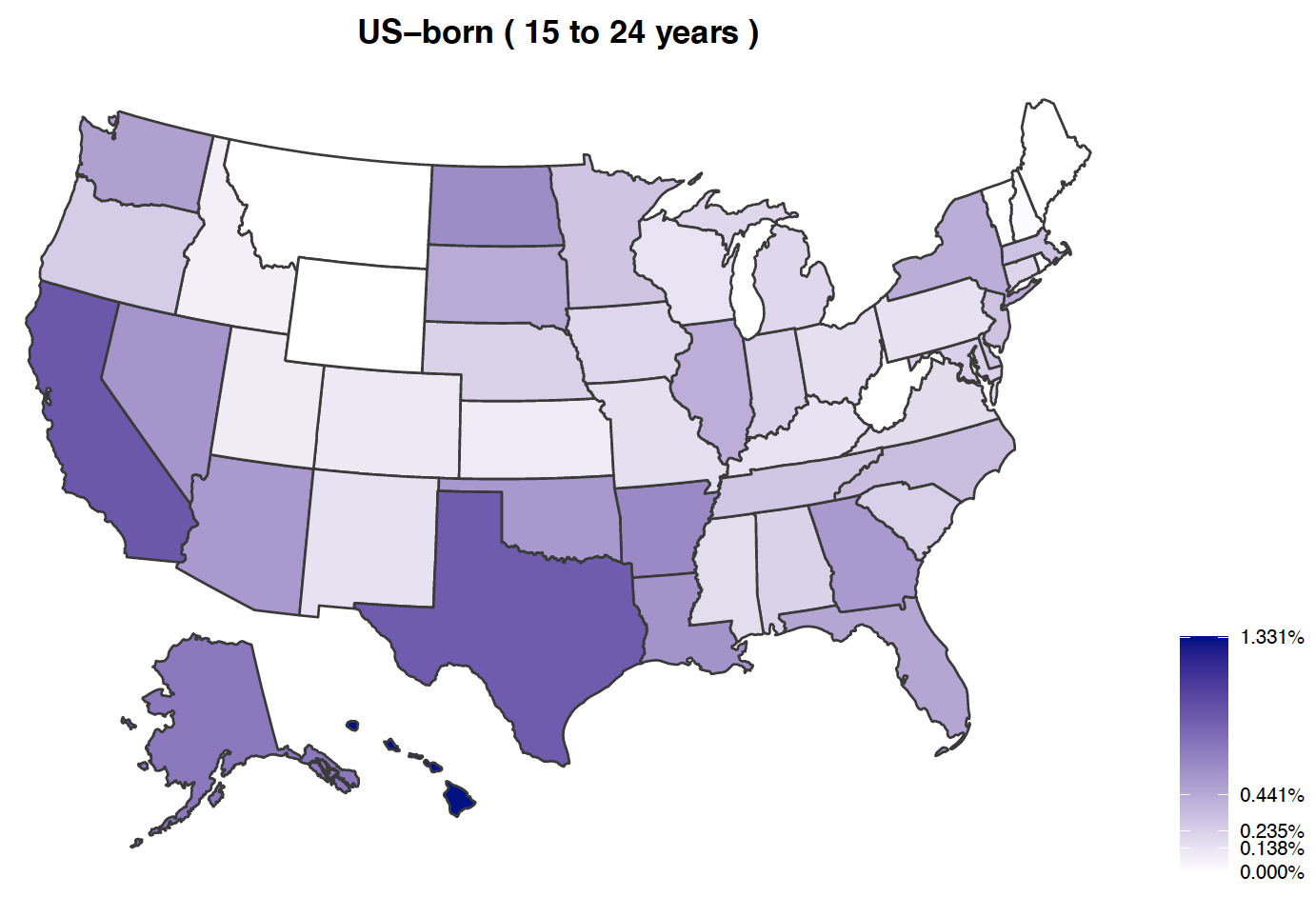** | 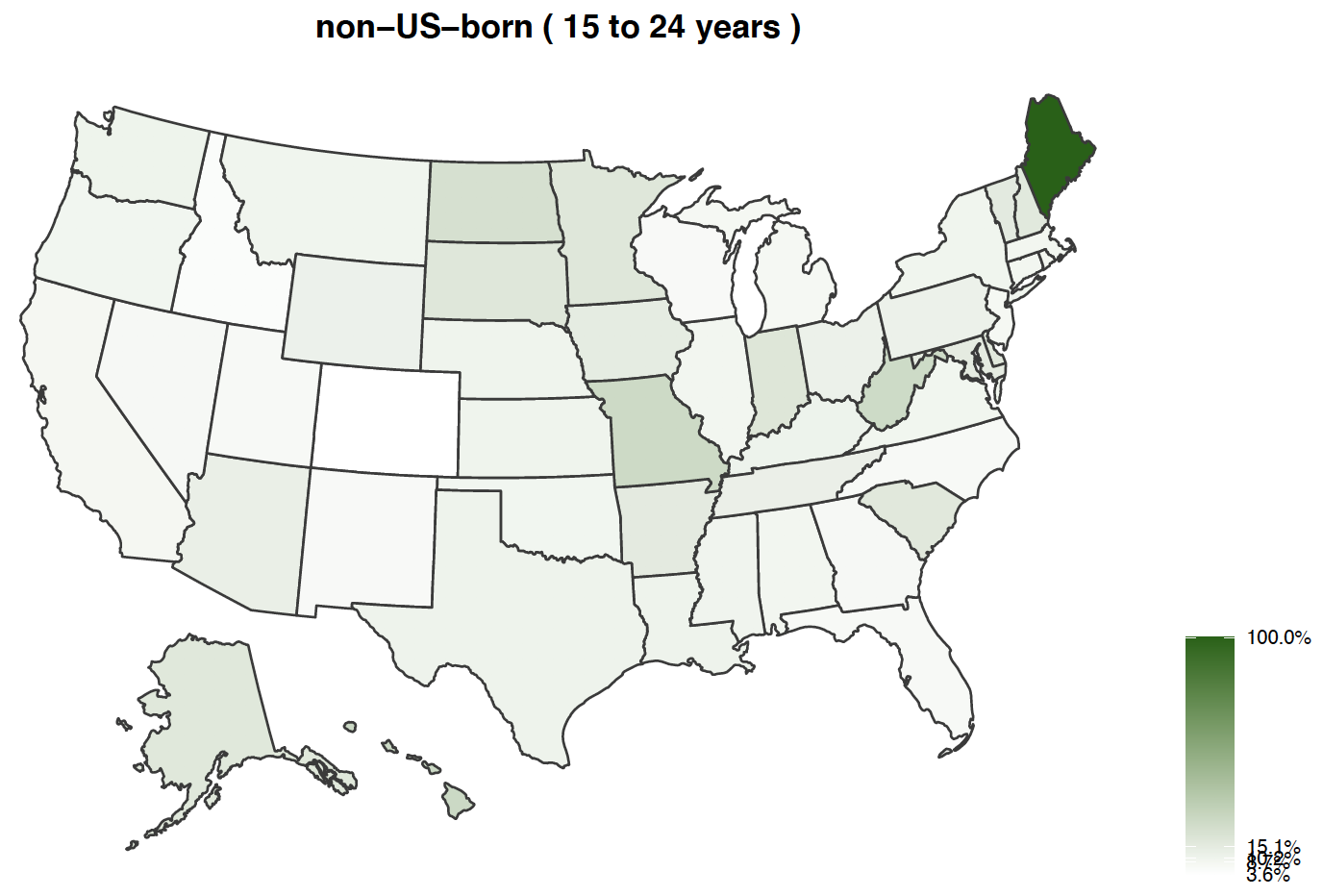 |
| **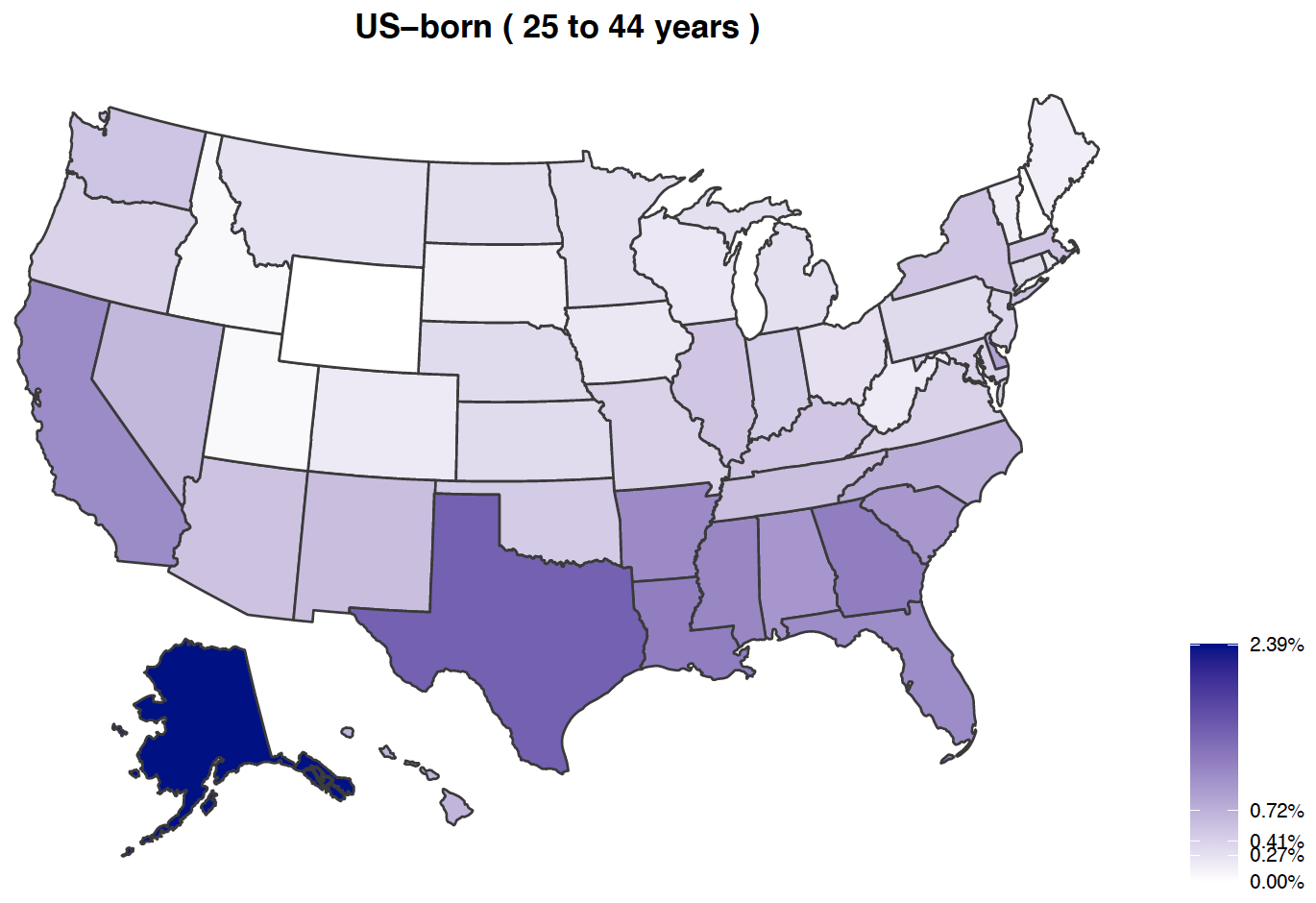** | 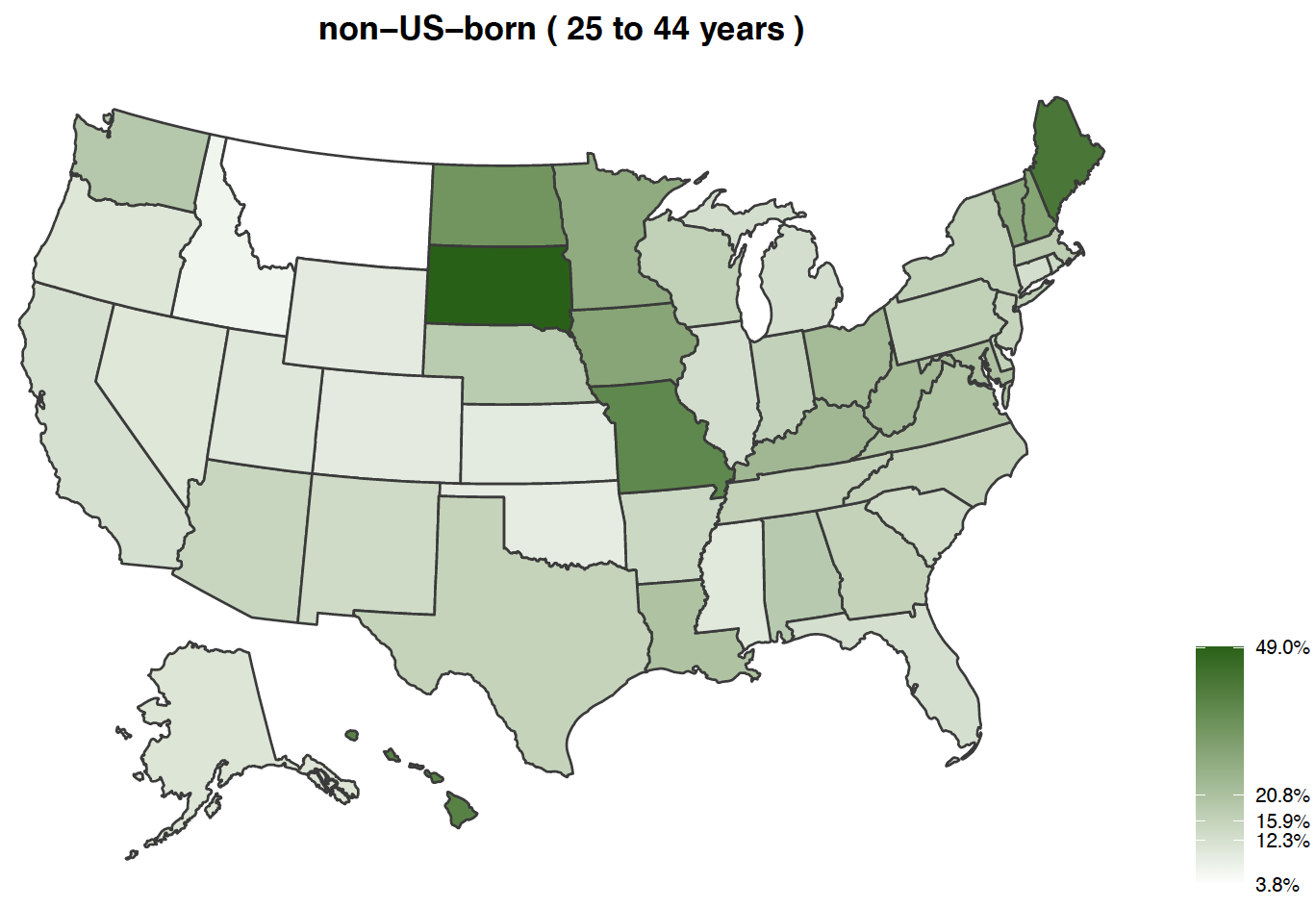 |

**S1 Figure. Continued**

| **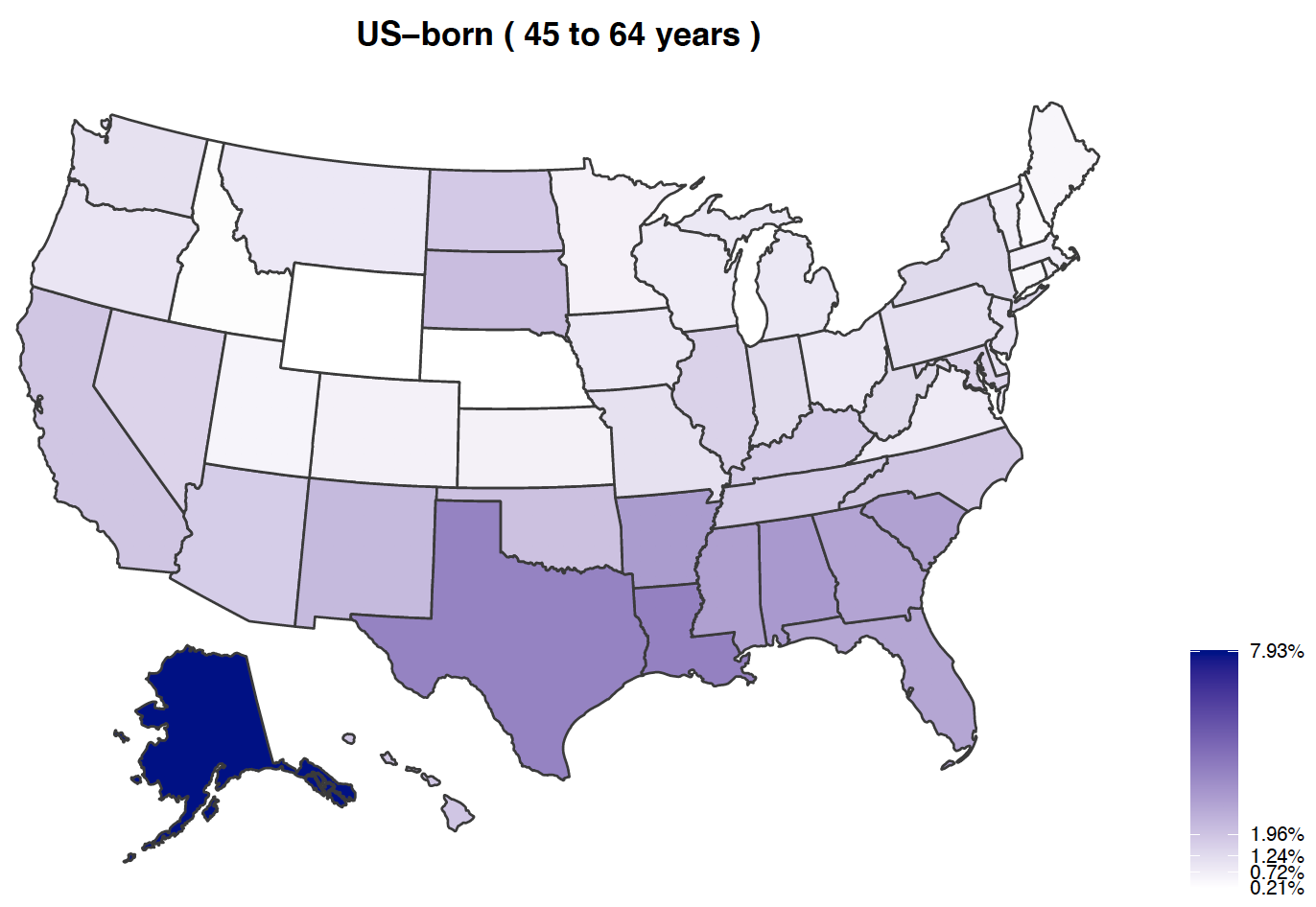** | 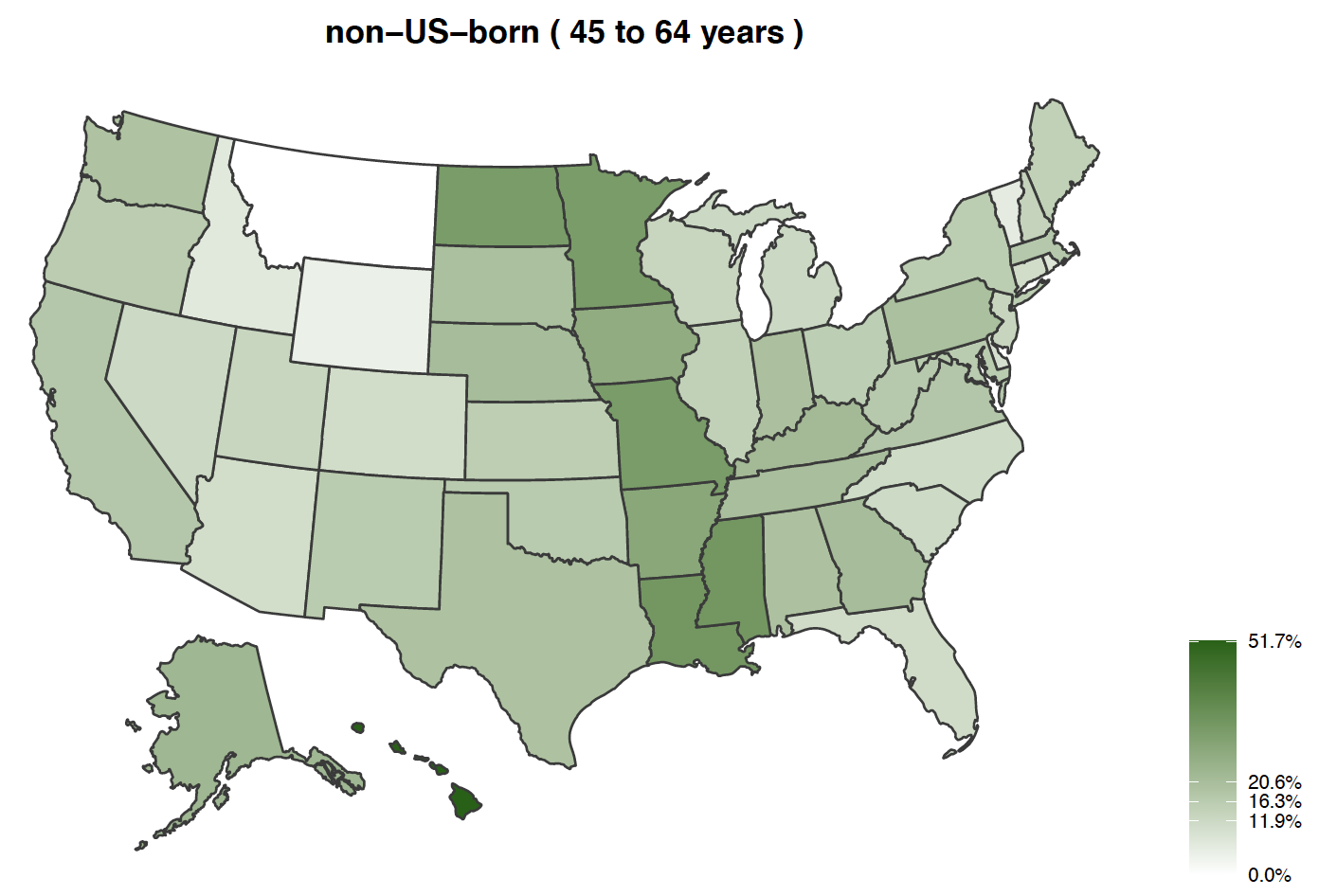 |
| --- | --- |
| **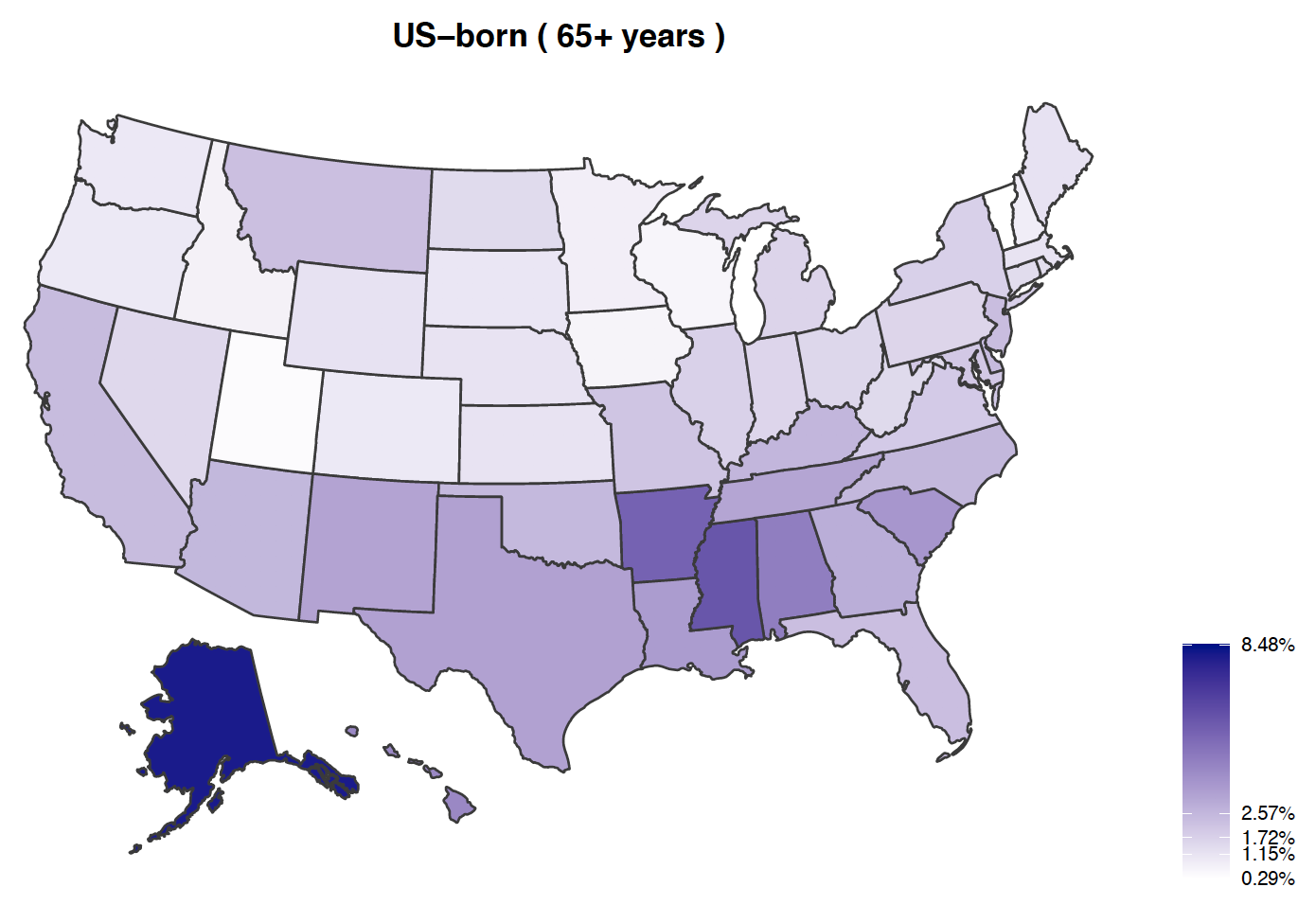** | 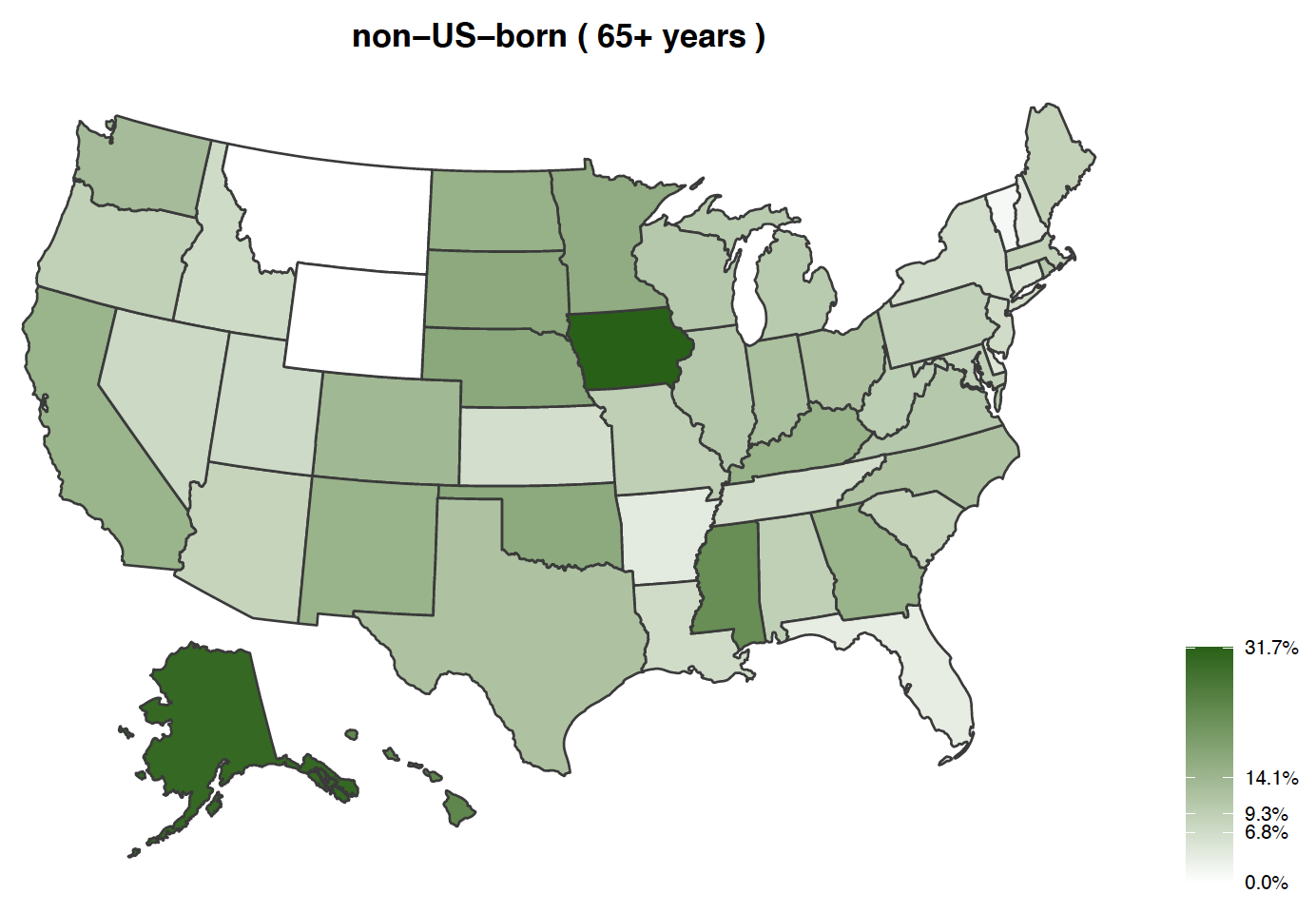 |

**S2 Figure. Estimated prevalence of latent tuberculosis infection by state and race/ethnicity, United States, 2015. (**The numbers in the legend are minimum, 25%, 50%, 75% quintiles, and maximum values.)*.* Software and source: open-source R and “usmap” package were used to create the maps. Both R software and the “usmap” package are license under GPL-3 | file LICENSE and free to use. [<https://www.r-project.org/Licenses/>; <https://cran.r-project.org/web/packages/usmap/usmap.pdf>]

| 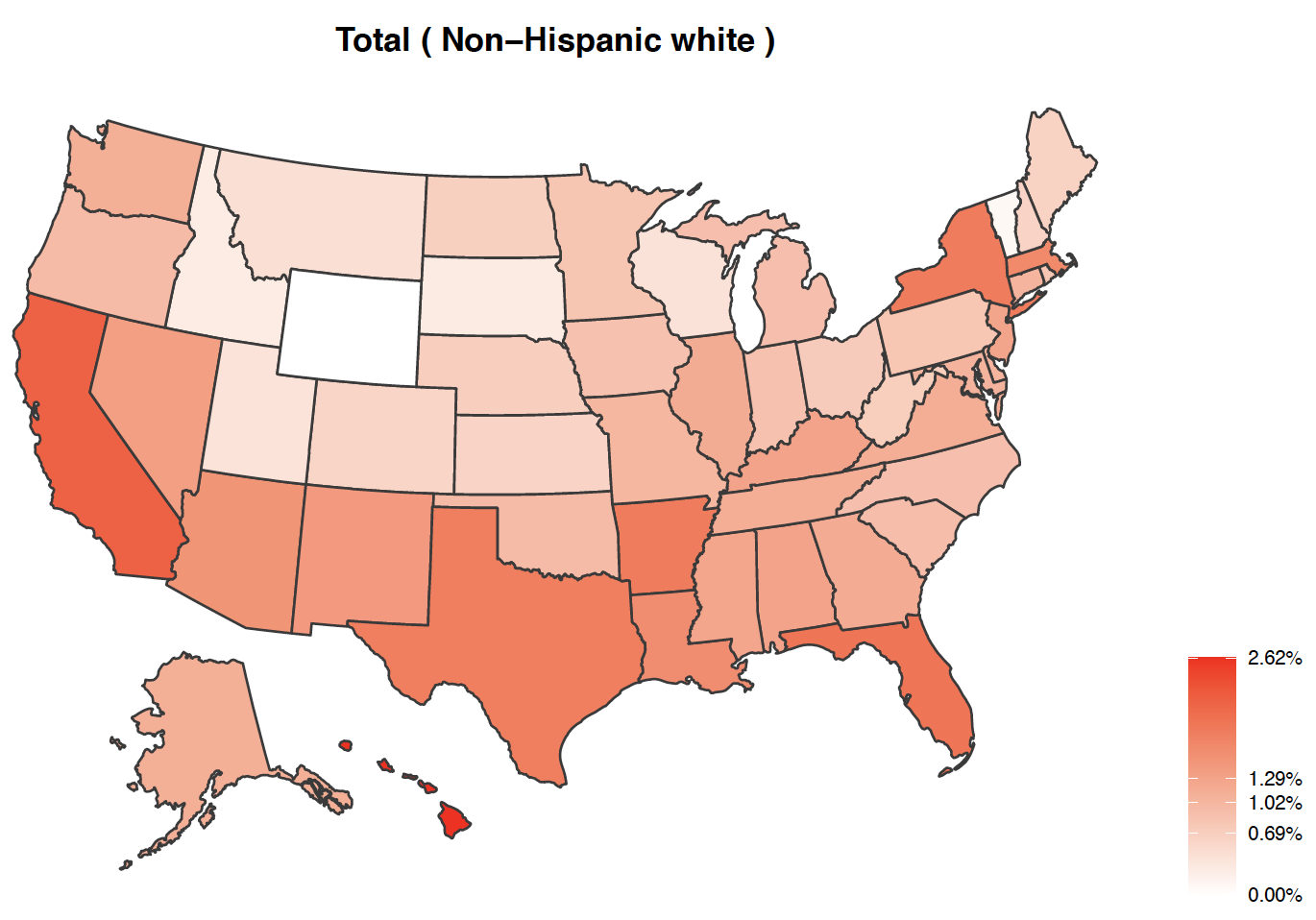 | 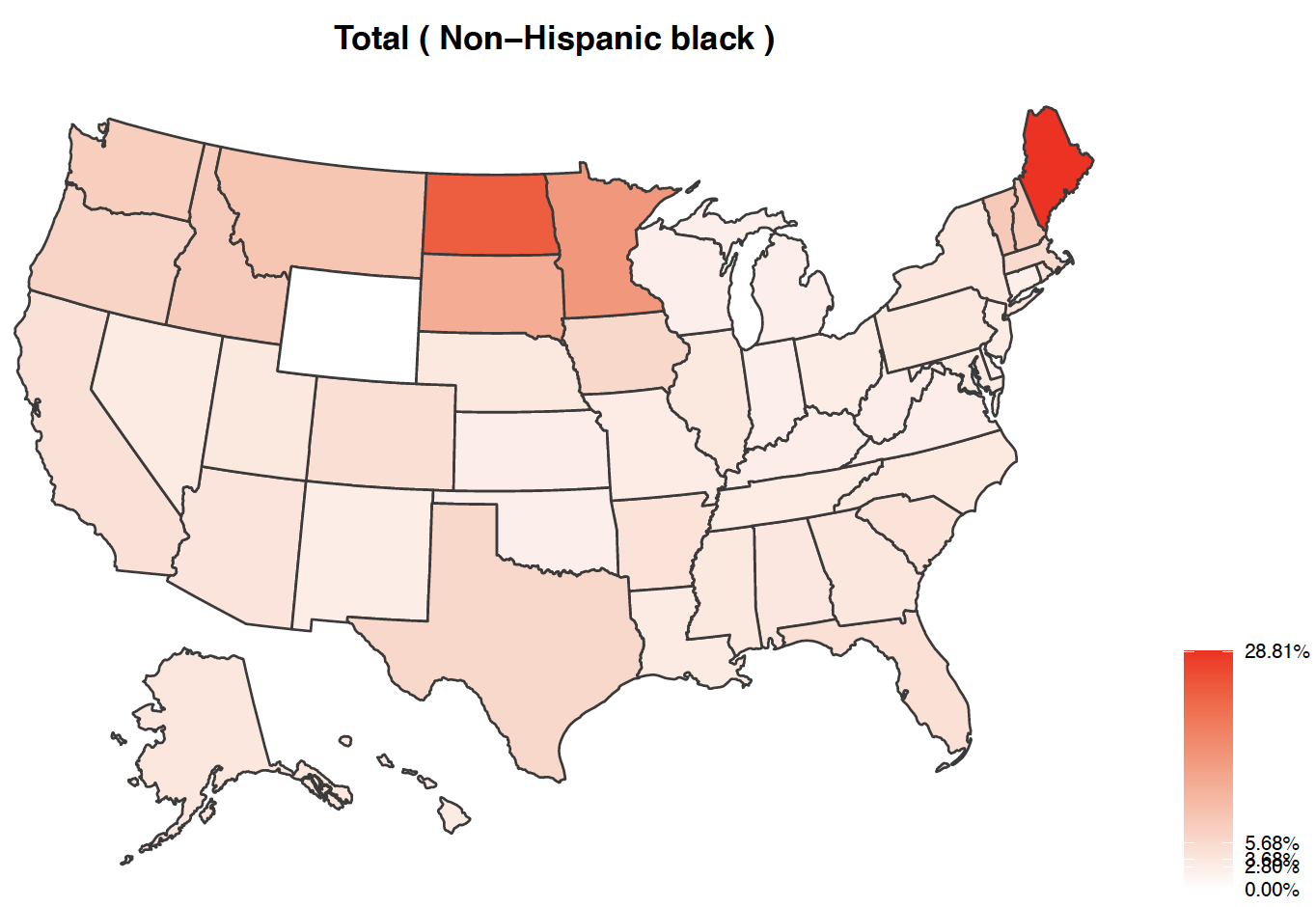 |
| --- | --- |
| **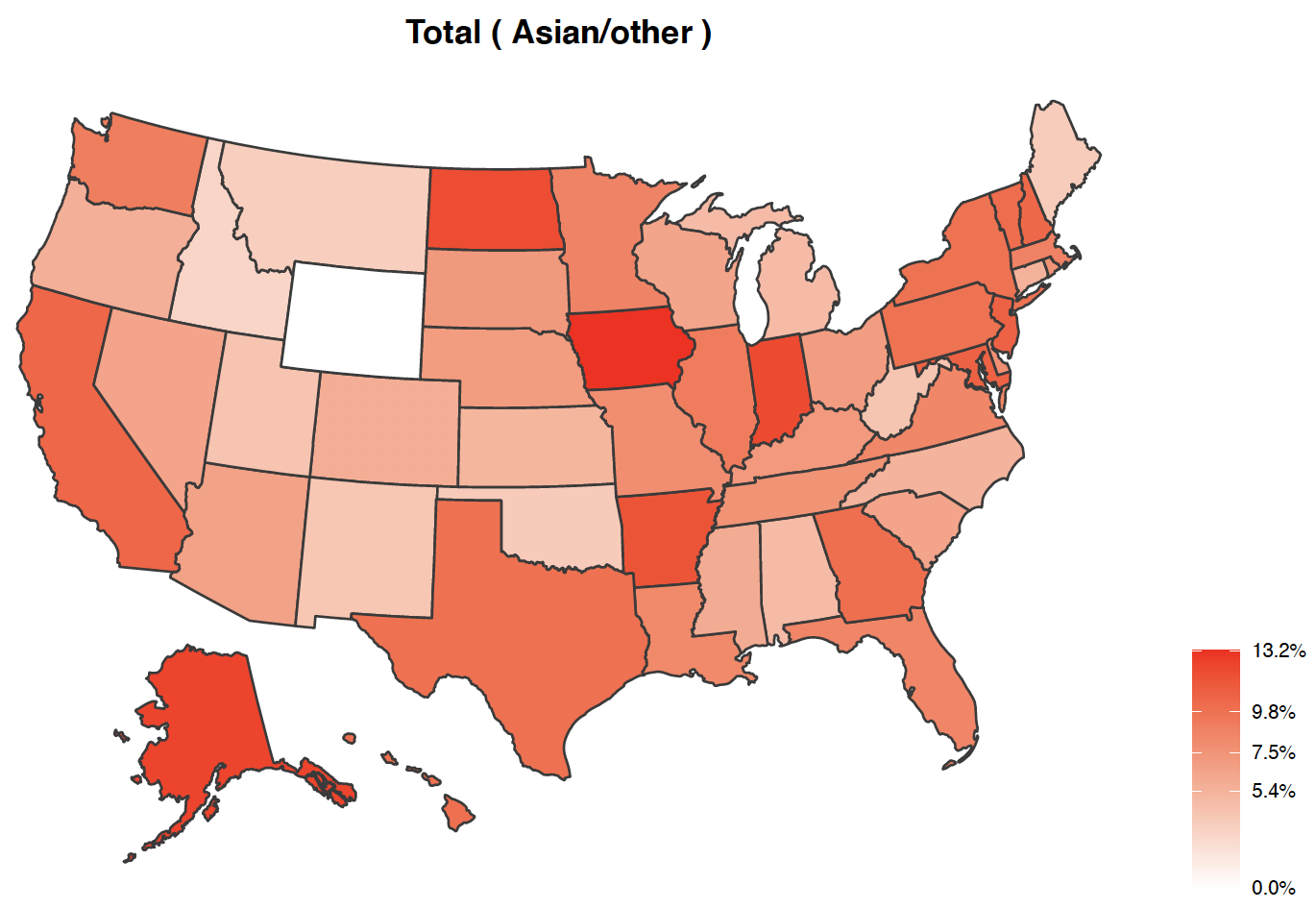** | 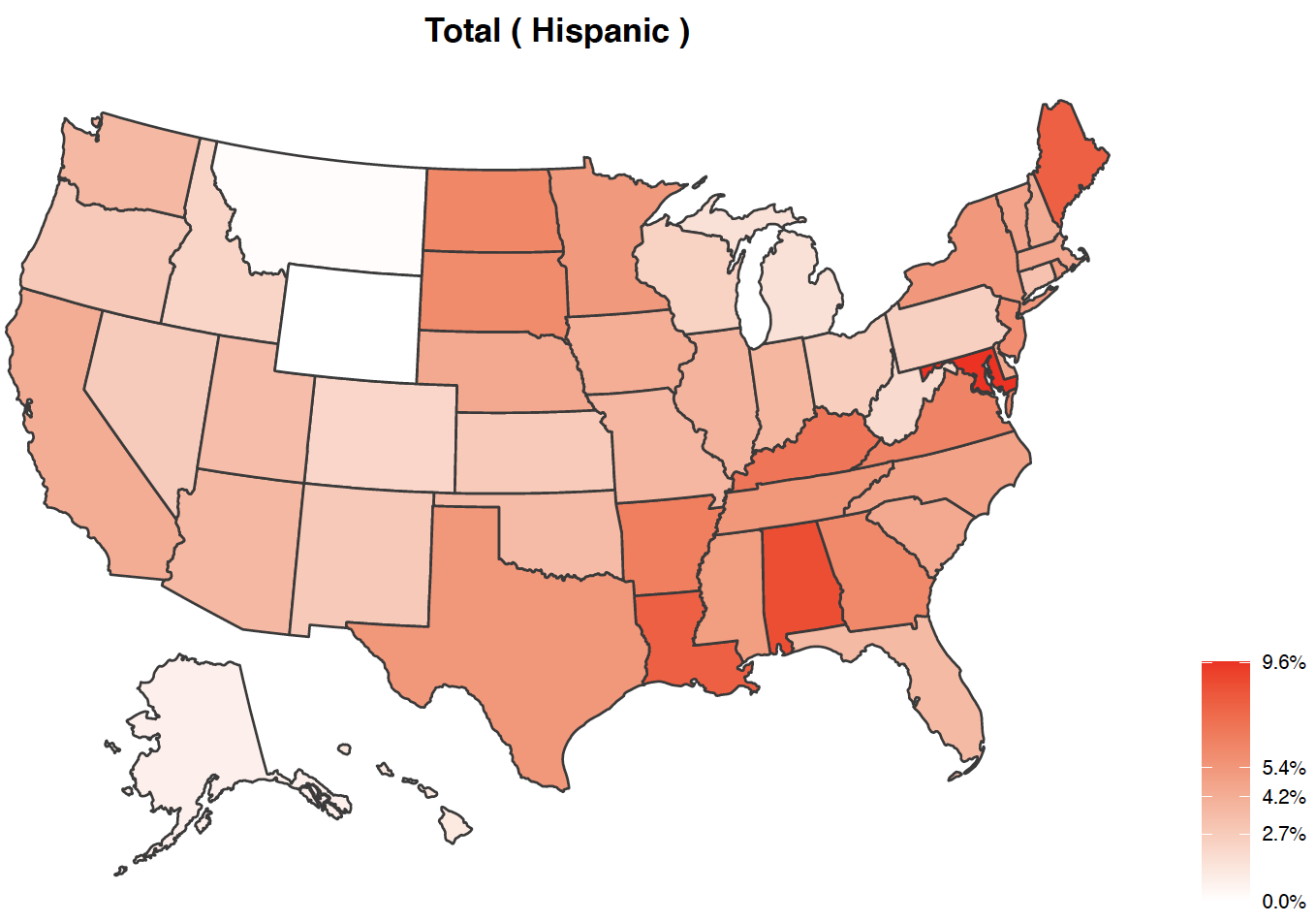 |
