# Supplementary material for: State-level prevalence estimates of latent tuberculosis infection in the United States by medical risk factors, demographic characteristics and nativity
Source: PLoS One. 2021 Apr 1;16(4):e0249012. doi: 10.1371/journal.pone.0249012 (PMC8016318; doi:10.1371/journal.pone.0249012)
Supplement: Attachment — Submitted filename: Response to Reviewers- PONE-D-20-36817R1.docx [file pone.0249012.s003.docx]

Journal Requirements:

When submitting your revision, we need you to address these additional requirements.

1. Please ensure that your manuscript meets PLOS ONE's style requirements, including those for file naming. The PLOS ONE style templates can be found at

https://journals.plos.org/plosone/s/file?id=wjVg/PLOSOne_formatting_sample_main_body.pdf and

https://journals.plos.org/plosone/s/file?id=ba62/PLOSOne_formatting_sample_title_authors_affiliations.pdf

**** Response:** we followed carefully the PLOS ONE's style requirements

2. We note that you have indicated that data from this study are available upon request. PLOS only allows data to be available upon request if there are legal or ethical restrictions on sharing data publicly. For information on unacceptable data access restrictions, please see http://journals.plos.org/plosone/s/data-availability#loc-unacceptable-data-access-restrictions.

In your revised cover letter, please address the following prompts:

a) If there are ethical or legal restrictions on sharing a de-identified data set, please explain them in detail (e.g., data contain potentially identifying or sensitive patient information) and who has imposed them (e.g., an ethics committee). Please also provide contact information for a data access committee, ethics committee, or other institutional body to which data requests may be sent.

b) If there are no restrictions, please upload the minimal anonymized data set necessary to replicate your study findings as either Supporting Information files or to a stable, public repository and provide us with the relevant URLs, DOIs, or accession numbers. Please see http://www.bmj.com/content/340/bmj.c181.long for guidelines on how to de-identify and prepare clinical data for publication. For a list of acceptable repositories, please see http://journals.plos.org/plosone/s/data-availability#loc-recommended-repositories.

We will update your Data Availability statement on your behalf to reflect the information you provide.

**** Response:** we added the following statement to the cover letter:

Data Availability: The data contain information abstracted from the national TB case report form called the Report of Verified Case of Tuberculosis (RVCT) (OMB No. 0920-0026). These data have been reported voluntarily to CDC by state and local health departments, and are protected under the Assurance of Confidentiality (Sections 306 and 308(d) of the Public Health Service Act, 42 U.S.C. 242k and 242m(d)), which prevents disclosure of any information that could be used to directly or indirectly identify patients. For more information, see the CDC/ATSDR Policy on Releasing and Sharing Data (at http://www.cdc.gov/maso/Policy/ReleasingData.pdf). A limited dataset is available at https://wonder.cdc.gov/tb.html. Researchers seeking additional data may apply to analyze National Tuberculosis Surveillance System data at CDC headquarters by contacting TBInfo@cdc.gov.

3. We note that Figure 1 and 2 and Supporting Information Figures S1 and S2 in your submission contain map images which may be copyrighted.

All PLOS content is published under the Creative Commons Attribution License (CC BY 4.0), which means that the manuscript, images, and Supporting Information files will be freely available online, and any third party is permitted to access, download, copy, distribute, and use these materials in any way, even commercially, with proper attribution. For these reasons, we cannot publish previously copyrighted maps or satellite images created using proprietary data, such as Google software (Google Maps, Street View, and Earth). For more information, see our copyright guidelines: http://journals.plos.org/plosone/s/licenses-and-copyright.

We require you to either (a) present written permission from the copyright holder to publish these figures specifically under the CC BY 4.0 license, or (b) remove the figures from your submission:

**** Response:** we used open-source R and “usmap” package to create the maps. Both R software and the “usmap” package are license under GPL-3 | file LICENSE and free to use.

- <https://www.r-project.org/Licenses/>
- <https://cran.r-project.org/web/packages/usmap/usmap.pdf>

a. You may seek permission from the original copyright holder of Figure 1 and 2 and Supporting Information Figures S1 and S2 to publish the content specifically under the CC BY 4.0 license.

We recommend that you contact the original copyright holder with the Content Permission Form ([http://journals.plos.org/plosone/s/file?id=7c09/content-permission-form.pdf](about:blank)) and the following text:

“I request permission for the open-access journal PLOS ONE to publish XXX under the Creative Commons Attribution License (CCAL) CC BY 4.0 ([http://creativecommons.org/licenses/by/4.0/](about:blank)). Please be aware that this license allows unrestricted use and distribution, even commercially, by third parties. Please reply and provide explicit written permission to publish XXX under a CC BY license and complete the attached form.”

Please upload the completed Content Permission Form or other proof of granted permissions as an "Other" file with your submission.

In the figure caption of the copyrighted figure, please include the following text: “Reprinted from [ref] under a CC BY license, with permission from [name of publisher], original copyright [original copyright year].”

b, If you are unable to obtain permission from the original copyright holder to publish these figures under the CC BY 4.0 license or if the copyright holder’s requirements are incompatible with the CC BY 4.0 license, please either i) remove the figure or ii) supply a replacement figure that complies with the CC BY 4.0 license. Please check copyright information on all replacement figures and update the figure caption with source information. If applicable, please specify in the figure caption text when a figure is similar but not identical to the original image and is therefore for illustrative purposes only.

The following resources for replacing copyrighted map figures may be helpful:

USGS National Map Viewer (public domain): [http://viewer.nationalmap.gov/viewer/](about:blank)

The Gateway to Astronaut Photography of Earth (public domain): [http://eol.jsc.nasa.gov/sseop/clickmap/](about:blank)

Maps at the CIA (public domain): [https://www.cia.gov/library/publications/the-world-factbook/index.html](about:blank) and [https://www.cia.gov/library/publications/cia-maps-publications/index.html](about:blank)

NASA Earth Observatory (public domain): [http://earthobservatory.nasa.gov/](about:blank)

Landsat: http://landsat.visibleearth.nasa.gov/

USGS EROS (Earth Resources Observatory and Science (EROS) Center) (public domain): [http://eros.usgs.gov/#](about:blank)

Natural Earth (public domain): [http://www.naturalearthdata.com/](about:blank)

4. Please include captions for your Supporting Information files at the end of your manuscript, and update any in-text citations to match accordingly. Please see our Supporting Information guidelines for more information: [http://journals.plos.org/plosone/s/supporting-information](about:blank)

**** Response:** we added the following section after the references, and also uploaded a new file for the Supporting Information:

**Supporting Information:**

Appendix 1: Methods to calculate the population-specific annual reactivation rates

Appendix 2: the analysis code in R

Appendix 3: the structure of “LTBI backcalc RR data.xlsx”

Appendix 4: The structure of tb_dummyRT.csv

Supplementary Table 1. Estimated prevalence and number of persons living with latent tuberculosis infection by medical risk factor, age group, and race/ethnicity groupings, stratified by nativity and in total population, in 50 US states and District of Columbia, 2015

Supplementary Figure 1. Estimated prevalence of latent tuberculosis infection by state and age, United States, 2015. (The numbers in the legend are minimum, 25%, 50%, 75% quintiles, and maximum values.)

Supplementary Figure 2. Estimated prevalence of latent tuberculosis infection by state and race/ethnicity, United States, 2015. (The numbers in the legend are minimum, 25%, 50%, 75% quintiles, and maximum values.)

Reviewers' comments:

Reviewer's Responses to Questions

**Comments to the Author**

1. Is the manuscript technically sound, and do the data support the conclusions?

The manuscript must describe a technically sound piece of scientific research with data that supports the conclusions. Experiments must have been conducted rigorously, with appropriate controls, replication, and sample sizes. The conclusions must be drawn appropriately based on the data presented.

Reviewer #1: Yes

Reviewer #2: Yes

2. Has the statistical analysis been performed appropriately and rigorously?

Reviewer #1: Yes

Reviewer #2: Yes

3. Have the authors made all data underlying the findings in their manuscript fully available?

The [PLOS Data policy](http://www.plosone.org/static/policies.action#sharing) requires authors to make all data underlying the findings described in their manuscript fully available without restriction, with rare exception (please refer to the Data Availability Statement in the manuscript PDF file). The data should be provided as part of the manuscript or its supporting information, or deposited to a public repository. For example, in addition to summary statistics, the data points behind means, medians and variance measures should be available. If there are restrictions on publicly sharing data—e.g. participant privacy or use of data from a third party—those must be specified.

Reviewer #1: Yes

Reviewer #2: Yes

4. Is the manuscript presented in an intelligible fashion and written in standard English?

PLOS ONE does not copyedit accepted manuscripts, so the language in submitted articles must be clear, correct, and unambiguous. Any typographical or grammatical errors should be corrected at revision, so please note any specific errors here.

Reviewer #1: Yes

Reviewer #2: Yes

5. Review Comments to the Author

Please use the space provided to explain your answers to the questions above. You may also include additional comments for the author, including concerns about dual publication, research ethics, or publication ethics. (Please upload your review as an attachment if it exceeds 20,000 characters)

Reviewer #1: PONE-D-20-36817
“State-level prevalence estimates of latent tuberculosis infection in the United States by medical risk factors, demographic characteristics and nativity”
Summary: This manuscript used previously published TB reactivation rates from the literature and back-calculated active tuberculosis cases to estimate current LTBI prevalence in the US. They examined these prevalences by age, race/ethnicity, a few selected comorbid conditions and nativity.

Major comments:
1. I think the method of trying to use active TB cases to back calculate LTBI prevalence is interesting though this manuscript estimated 8.6 million of Americans having LTBI and this is substantially lower than what NHANES survey estimated (13.3 million). Where do you think the large discrepancy is coming from?

**** Response:** thank you for the valuable comment. As suggested, we compared our estimates with those from NHANES survey 2011-12, and added the following section the discussion section:

Compared to the LTBI prevalence estimates from NHANES 2011-12 (4.7% total, 1.5% US-born, 20.5% non-US-born) (Ref. Miramontes et al Plos One 2015), our results were much lower for total (2.7%) and non-US-born (13.9%). The estimates from NHANES were based on Tuberculin skin test (TST) results and thus might have overestimated true LTBI prevalence due to cross reaction with BCG vaccine. When Interferon Gamma Release Assay (IGRA) positivity was used in NHANES, the LTBI estimate for non-US-born dropped to 15.9% (95%CI 13.5 – 18.7) (Ref. Miramontes et al Plos One 2015), much closer to our estimate. However, our estimates for LTBI among older non-US-born groups was higher than NHANES IGRA findings: age 45-64 years (23.5% vs. 16.3%) and 65+ years (32.1% vs 9.8%). This may reflect cohort effects, with 2010 older birth cohorts (with higher TB infection rates) being replaced in recent years with lower infection rate younger birth cohorts (Ref. Winston CA BMC 2010).

- Miramontes R, Hill AN, Yelk Woodruff RS, Lambert LA, Navin TR, et al. (2015) Tuberculosis Infection in the United States: Prevalence Estimates from the National Health and Nutrition Examination Survey, 2011-2012. PLOS ONE 10(11): e0140881
- Winston CA, Navin TR. [1]. 2010 Jul 13;10:206. doi: 10.1186/1471-2334-10-206. PMID: 20626871; PMCID: PMC2915994.

NHANES survey estimates for LTBI in 2011-12 as reported by Miramontes et al 2015:

Our LTBI estimation for 2015:

2. I think this manuscript and the data and results presented are intriguing. However, it is adding only a few additional components with limitations to the analyses by Shea et al and Haddad et al. For example, this analysis included comorbid conditions but significant assumptions had to be made when deriving the prevalence of comorbid conditions (from the active TB cases) including whether TB came before the comorbid conditions. Also, the model doesn’t take into account treatment for LTBI and if you are arguing that these estimates could be use to target testing and treating, this is big point.

**** Response:** We appreciate the positive feedback. As you mention above, our analysis had some limitations which we recognized and discussed them in the paper.

3. I think generally there is agreement that you should screen those who were not born in the US for LTBI and then consider treatment. I am not sure that this manuscript adds evidence one way or another to the treatment question.

**** Response:** we agree with the limitation of our study regarding the message about LTBI treatment. The main focus of our paper was to produce national and state-level estimates of LTBI prevalence, both in the total population and within sub-groups.

4. I think it is admirable to include the other comorbidities but they are included as only what comorbidity at a time. Also the immunosuppressive therapy category is huge but what about the disease modifying agents for all of the rheumatologic conditions? Also what about malignancies?

**** Response:** We acknowledged the limitations of our analysis in estimating the LTBI by comorbidities (i.e., medical risk factor) in the discussion. This limitation was imposed by the type and details of comorbidities of TB cases which reported to the CDC National Tuberculosis Surveillance System. Also, we used literature review to collect the reactivation rate ratios (RRRs) and the RRRs were not reported for all medical risk factor, including for cancer:

For TB cases with more than one medical risk factor, we considered only the risk factor with the higher reactivation rate. This hierarchy might have led to an overestimation of the LTBI prevalence by medical risk factor if actually having multiple risk factors would cause an even higher reactivation risk. This limitation also prevented us from being able to provide more refined LTBI prevalence estimates for persons with multiple medical comorbidities.

5. I wonder if you could include treatment with isoniazid into the model to try and refine the estimates and include some of the treatment for LTBI.

**** Response:** Our back-calculation model did not require the data on treatment for LTBI as an input. As we stated in the discussion section, our analysis did not assess the effect of LTBI treatment; however, it is likely that treated LTBI would not have reactivated to TB disease and so were automatedly excluded from our back-calculation. So, one simple way to account for those LTBI cases who were found and treated by isoniazid would be adding their numbers to our estimates for LTBI. We did not have access to LTBI treated data at national and subnational, and by population subgroups, and so we decided not to do this post-HOC adjustment.

Minor comments:
1. I’m having a hard time reconciling that person of Asian or other race/ethnicity in the total US population had the highest LTBI prevalence, but then in the next paragraph it said that most of the persons estimated as having LTBI were non-Hispanic white.

**** Response:** We think you are referring to the following sentences of the result section. The data supporting these are presented in Tables 4 and 2.

Persons of Asian or other race/ethnicity in the total U.S. population were estimated to have the highest LTBI prevalence (8.7%). [see Table 4 please]

Most of the US-born persons estimated as having LTBI were non-Hispanic White (46.5%). [see Table 2 please]

2. When describing the state prevalence of LTBI, I’m not sure how informative describe LTBI prevalence >1% and then in 26 states it was over 15% in non-US born. Are there reasons for describing these cut-offs?

**** Response:** We agree that choosing a cut-off is challenging. In text, we choose the cut-offs of 1% for US-born population, and 15% non-US–born population to summaries the number of stated that are more than the national average estimates. In Figure 1, our color scheme and legend were chosen based on minimum, 25%, 50%, 75% quintiles, and maximum values for each sub-population.

Reviewer #2: The manuscript entitled “State-level prevalence estimates of latent tuberculosis infection in the United States by medical risk factors, demographic characteristics and nativity”. Overall, the manuscript is well organized.

**** Response:** We appreciate the positive feedback.

General comments
The study is interesting and gives good figure on LTBI status in US. However, I have the following concerns.
1. The study is talking about prevalence of LTBI in 2015. Now it is 2020. What is the relevant of your study on today epidemiology as well as prevention and control TB in US?

**** Response:** this is a valuable comment. As we mentioned in the method section, we used TB data from 2013 to 2017 for our analysis, and then reported the average estimates for this period as LTBI estimates for 2015. At the time of analysis, the TB data were available up to 2017.

2. As you mentioned in your rationale, study on prevalence of LTBI in US was conducted from 2011 to 2015 by Haddad et al. In addition, in 2nd paragraph of your discussion you mentioned as LTBI national survey was conducted in US and high prevalence of LTBI was reported among non-US born. Moreover, in the same paragraph you mentioned high LTBI in California. Concerning these, again what is the relevance of your study to US?

**** Response:** The main focus of our analysis was to produce national and state-level estimates of LTBI prevalence, both in the total population and within sub-groups. None of these estimates were previously estimated and reported.

Specific comments
1. In result part under sub-title characteristics of personals predicted to have LTBI: Remove the word few from the initial of two paragraphs because even the percentages are low the actual numbers are more than hundred thousand.

**** Response:** Good point. We revised accordingly.

2. You mentioned about seven limitations and three advantages of your study. How you comment on this? On the top, one of your advantages was having similar result with the previous two studies in the area. How come having similar finding become advantage of your study?

**** Response:** In the limitation section, we did our best to clearly mention all major limitations of our analysis and explained their effects on our LTBI estimates. We agree with you and so dropped “the similar result with the previous two studies” from the advantages of our study.

Final decision: The manuscript is accepted for publication with minor revision

**** Response:** We appreciate the positive feedback.

6. PLOS authors have the option to publish the peer review history of their article ([what does this mean?](https://journals.plos.org/plosone/s/editorial-and-peer-review-process#loc-peer-review-history)). If published, this will include your full peer review and any attached files.


If you choose “no”, your identity will remain anonymous but your review may still be made public.


**Do you want your identity to be public for this peer review?** For information about this choice, including consent withdrawal, please see our [Privacy Policy](https://www.plos.org/privacy-policy).

Reviewer #1: No

Reviewer #2: No

[NOTE: If reviewer comments were submitted as an attachment file, they will be attached to this email and accessible via the submission site. Please log into your account, locate the manuscript record, and check for the action link "View Attachments". If this link does not appear, there are no attachment files.]

While revising your submission, please upload your figure files to the Preflight Analysis and Conversion Engine (PACE) digital diagnostic tool, <https://pacev2.apexcovantage.com/>. PACE helps ensure that figures meet PLOS requirements. To use PACE, you must first register as a user. Registration is free. Then, login and navigate to the UPLOAD tab, where you will find detailed instructions on how to use the tool. If you encounter any issues or have any questions when using PACE, please email PLOS at [figures@plos.org](mailto:figures@plos.org). Please note that Supporting Information files do not need this step.

*In compliance with data protection regulations, you may request that we remove your personal registration details at any time.* [*(Remove my information/details)*](https://www.editorialmanager.com/pone/login.asp?a=r)*. Please contact the publication office if you have any questions.*

1.	Winston CA, Navin TR. Birth cohort effect on latent tuberculosis infection prevalence, United States. BMC Infect Dis. 2010;10:206. Epub 2010/07/16. doi: 10.1186/1471-2334-10-206. PubMed PMID: 20626871; PubMed Central PMCID: PMCPMC2915994.
